# Novel Miscanthus Germplasm-Based Value Chains: A Life Cycle Assessment

**DOI:** 10.3389/fpls.2017.00990

**Published:** 2017-06-08

**Authors:** Moritz Wagner, Andreas Kiesel, Astley Hastings, Yasir Iqbal, Iris Lewandowski

**Affiliations:** ^1^Department Biobased Products and Energy Crops, Institute of Crop Science, University of HohenheimStuttgart, Germany; ^2^The School of Biological Sciences, University of AberdeenAberdeen, United Kingdom

**Keywords:** miscanthus, biobased value chains, LCA, environmental performance, normalization, impact categories

## Abstract

In recent years, considerable progress has been made in miscanthus research: improvement of management practices, breeding of new genotypes, especially for marginal conditions, and development of novel utilization options. The purpose of the current study was a holistic analysis of the environmental performance of such novel miscanthus-based value chains. In addition, the relevance of the analyzed environmental impact categories was assessed. A Life Cycle Assessment was conducted to analyse the environmental performance of the miscanthus-based value chains in 18 impact categories. In order to include the substitution of a reference product, a system expansion approach was used. In addition, a normalization step was applied. This allowed the relevance of these impact categories to be evaluated for each utilization pathway. The miscanthus was cultivated on six sites in Europe (Aberystwyth, Adana, Moscow, Potash, Stuttgart and Wageningen) and the biomass was utilized in the following six pathways: (1) small-scale combustion (heat)—chips; (2) small-scale combustion (heat)—pellets; (3) large-scale combustion (CHP)—biomass baled for transport and storage; (4) large-scale combustion (CHP)—pellets; (5) medium-scale biogas plant—ensiled miscanthus biomass; and (6) large-scale production of insulation material. Thus, in total, the environmental performance of 36 site × pathway combinations was assessed. The comparatively high normalized results of human toxicity, marine, and freshwater ecotoxicity, and freshwater eutrophication indicate the relevance of these impact categories in the assessment of miscanthus-based value chains. Differences between the six sites can almost entirely be attributed to variations in biomass yield. However, the environmental performance of the utilization pathways analyzed varied widely. The largest differences were shown for freshwater and marine ecotoxicity, and freshwater eutrophication. The production of insulation material had the lowest impact on the environment, with net benefits in all impact categories expect three (marine eutrophication, human toxicity, agricultural land occupation). This performance can be explained by the multiple use of the biomass, first as material and subsequently as an energy carrier, and by the substitution of an emission-intensive reference product. The results of this study emphasize the importance of assessing all environmental impacts when selecting appropriate utilization pathways.

## Introduction

The developing European bioeconomy will lead to an increasing demand for sustainably produced biomass in the near future. Miscanthus is one of the leading candidate biomass crops and has the advantage that it can also grow under marginal site conditions (Lewandowski et al., 2016). It is a perennial rhizomatous C4 grass originating from Southeast Asia, where it shows large genetic diversity. Miscanthus was introduced into Europe in 1935, where the genotype *Miscanthus* × *giganteus* is predominately cultivated (Clifton-Brown et al., 2015). It is a resource-efficient, low-input crop, which can achieve yields of well above 20 Mg ha^−1^ a^−1^ (dry matter) in Central Europe (Lewandowski and Schmidt, 2006; Iqbal et al., 2015) and more than 30 Mg ha^−1^ a^−1^ (dry matter) in southern Europe under irrigated conditions (Lewandowski et al., 2000). As a perennial crop, miscanthus can be harvested over a 15–20-year cultivation period (Lewandowski et al., 2000; Christian et al., 2008). Due to its perennial nature and its high nitrogen- and water-use efficiency, miscanthus has a comparatively low impact on the environment as a biomass crop (Lewandowski et al., 2000; Voigt, 2015; McCalmont et al., 2017).

Miscanthus biomass can be used in several different utilization pathways. When harvested green in the period September to October, it can be used as a biogas substrate (Whittaker et al., 2016; Kiesel and Lewandowski, 2017). When harvested in early spring, it is suitable for combustion (Dahl and Obernberger, 2004; Iqbal and Lewandowski, 2014), as a late harvest leads to a lower water and mineral content (Lewandowski et al., 2000). In addition, miscanthus biomass can be fermented to ethanol (van der Weijde et al., 2016) or used as a raw material for the production of insulation material (Uihlein et al., 2008) or bio-composites (Muthuraj et al., 2015).

However, despite these diverse potential applications, there is currently low implementation of miscanthus cultivation as several major barriers hinder its utilization in practice (Clifton-Brown et al., 2016). To overcome these barriers, considerable efforts have been made in the last years in (a) development of new genotypes, tailored to different, especially marginal, site conditions in Europe, and different biomass uses; (b) the optimization of miscanthus management (Clifton-Brown et al., 2016; Lewandowski et al., 2016).

The objective of this study is to assess the environmental performance of various miscanthus-based energetic and material value chains using the most up-to-date genotype as well as management options. Most previous studies used cultivation and yield data from the standard genotype *Miscanthus* × *giganteus* to analyse environmental performance. However, as explained above, in the last years there have been substantial efforts especially in the breeding of new genotypes. The inclusion of this progress in the current study will allow a more realistic assessment of the environmental impact and mitigation possibilities of miscanthus-based value chains.

Several studies have already evaluated the environmental performance of miscanthus-based value chains in different impact categories. These studies encompass the utilization of miscanthus as a biogas substrate (Kiesel et al., 2016), for electricity generation (Sanscartier et al., 2014), as feedstock for bioethanol (Jeswani et al., 2015), and as fuel for heat generation (Wagner and Lewandowski, 2017). However, most of these studies examine only one single utilization pathway or assess only a few impact categories (Meyer et al., 2016).

The various assumptions, system boundaries and methodologies used in these studies makes a comparison of the results very difficult. Therefore, the second objective of the current study is to assess the environmental sustainability of different miscanthus utilization pathways in several impact categories under the same assumptions and underlying conditions. This is done in order to enable the comparison of the environmental performance of different miscanthus-based value chains.

For this purpose, an attributional Life Cycle Assessment (LCA) was conducted according to the ISO standards 14040 and 14044 (ISO, 2006a,b). The energetic and material utilization pathways assessed in this study are: (1) small-scale combustion (heat)—chips; (2) small-scale combustion (heat)—pellets; (3) large-scale combustion (CHP)—biomass baled for transport and storage; (4) large-scale combustion (CHP)—pellets; (5) medium-scale biogas plant—biomass ensiled; and (6) large-scale production of insulation material—biomass baled for transport and storage. These pathways were assessed for miscanthus biomass cultivated from different genotypes on six climatically different sites across Europe: Aberystwyth (UK), Adana (Turkey), Moscow (Russia), Potash (Ukraine), Stuttgart (Germany), and Wageningen (Netherlands). Data for the cultivation of the biomass were provided through the EU-funded research project OPTIMISC (Optimizing Miscanthus Biomass Production) (Lewandowski et al., 2016). The environmental performance of each of the six utilization pathways was assessed for each site in 18 impact categories using the life-cycle impact assessment methodology ReCiPe (Goedkoop et al., 2008). To assess the mitigation potential of the analyzed pathways in the different impact categories, a system expansion approach was chosen. This approach enabled the assessment of the net benefits and impacts of the different pathways on the environment through the substitution of a chiefly fossil-based reference product with a miscanthus-based one.

In addition, a normalization step was applied. This allows the relevance of the analyzed impact categories for each utilization pathway to be assessed (Wagner and Lewandowski, 2017). The normalization factors used in this study were taken from the ReCiPe methodology (Goedkoop et al., 2008).

## Materials and methods

### Scope and boundaries

The scope of this study is a cradle-to-grave analysis of the environmental performance of miscanthus cultivation at six sites in Europe and the subsequent utilization in six pathways. In total, 36 site × pathway combinations were assessed. In order to include the substitution of a reference product, a system expansion approach was applied. This allows the impact of the substitution of a reference product (e.g., heat produced by the combustion of natural gas) through the utilization of 1 ha miscanthus (e.g., heat produced by the combustion of miscanthus chips) to be included in the assessment for each value chain. Thus, negative values represent burdens avoided by such a substitution, while positive values represent an additional impact through the use of miscanthus biomass. This is the case when the production and utilization of the reference products emits less than the substituting miscanthus-based product.

The functional unit (FU) as well as main and co-products for the six utilization pathways are shown in Table 1. In addition, for each product, the substituted reference product is indicated. One hectare was chosen as functional unit to assess the annual net benefit or impact of substituting a reference product by the energetic or material utilization of miscanthus. On the cultivation sites Aberystwyth (UK), Moscow (Russia), Potash (Ukraine), Stuttgart (Germany), and Wageningen (Netherlands), the genotype OPM-06 was used, a *M. sinensis* × *M. sacchariflorus* hybrid. On the Adana site in Turkey, the genotype *M* × *giganteus* (OPM-09) was used. These two were preselected from 15 assessed genotypes, because they were the most suitable for the location and utilization pathway in terms of biomass quality and yield. The data on the cultivation process and choice of genotypes are based on multi-location field trials described in Lewandowski et al. (2016). The sites in Adana, Potash, Stuttgart and Wageningen are mostly on land previously used as agricultural land, whereas the sites in Aberystwyth and Moscow are on marginal land. In Aberystwyth, the miscanthus was cultivated on land which was previously low-quality grassland. At the Moscow site, harsh winters lead to non-ideal growing conditions (Lewandowski et al., 2016).

**Table 1:** Utilization pathways assessed in this study, the functional unit, their outputs and the reference products.

**No**.	**Utilization pathway**	**Biomass used**	**FU**	**Output**	**Main product**	**Co-product**	**Reference product**
1	Small-scale combustion	Chips	1 ha	Heat	^*^		Heat produced by combustion of light fuel oil
2	Small-scale combustion	Pellets	1 ha	Heat	^*^		Heat produced by combustion of light fuel oil
3	Large-scale combustion (CHP)	Bales	1 ha	Heat	^*^		Heat produced by combustion of natural gas in a CHP
				Electricity		^*^	European electricity mix
4	Large-scale combustion (CHP)	Pellets	1 ha	Heat	^*^		Heat produced by combustion of natural gas in a CHP
				Electricity		^*^	European electricity mix
5	Biogas plant	Silage	1 ha	Electricity	^*^		European electricity mix
				Heat		^*^	Heat produced by combustion of natural gas in a CHP
6	Production of insulation material	Bales	1 ha	Insulation material	^*^		Glass wool

* *Indicates if the product is the main- or the co-product*.

**Figure 1:**
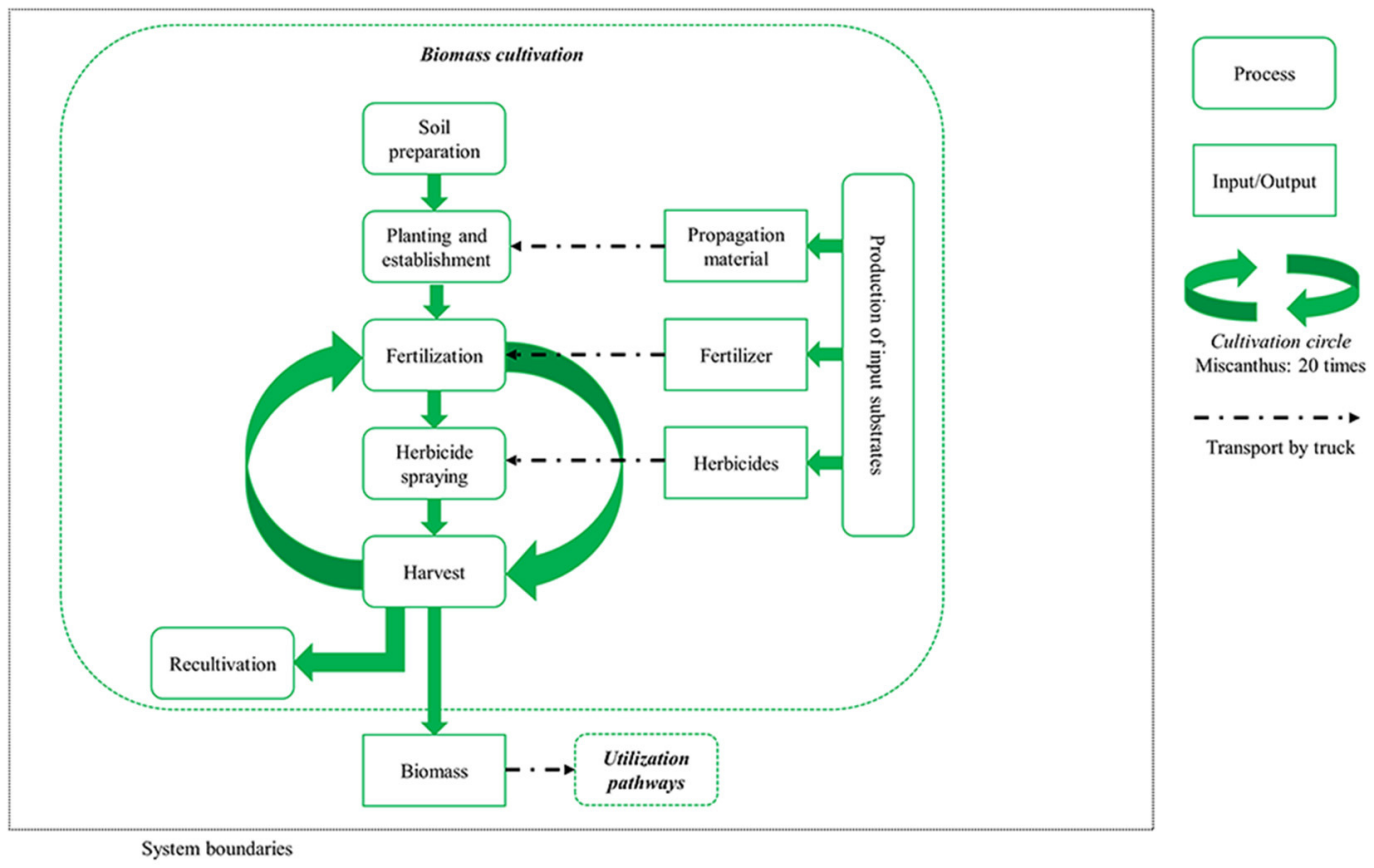
System description and boundaries for miscanthus biomass cultivation.

The agricultural system is described in Figure 1. The system boundaries include the production of input substrates (e.g., fertilizers, propagation material) and the whole cultivation process (from soil preparation through planting and establishment to harvest over a twenty-year cultivation period) to subsequent recultivation. For all utilization pathways, the miscanthus is mulched in the first year and harvested from the second year onwards. In pathways 2, 3, 4, and 6, it is mowed and then pressed into bales; in 1 and 5 it is harvested with a self-propelled forage harvester in the form of chips. For the combustion pathways 2 and 4, the miscanthus bales are then further processed to pellets.

**Figure 2:**
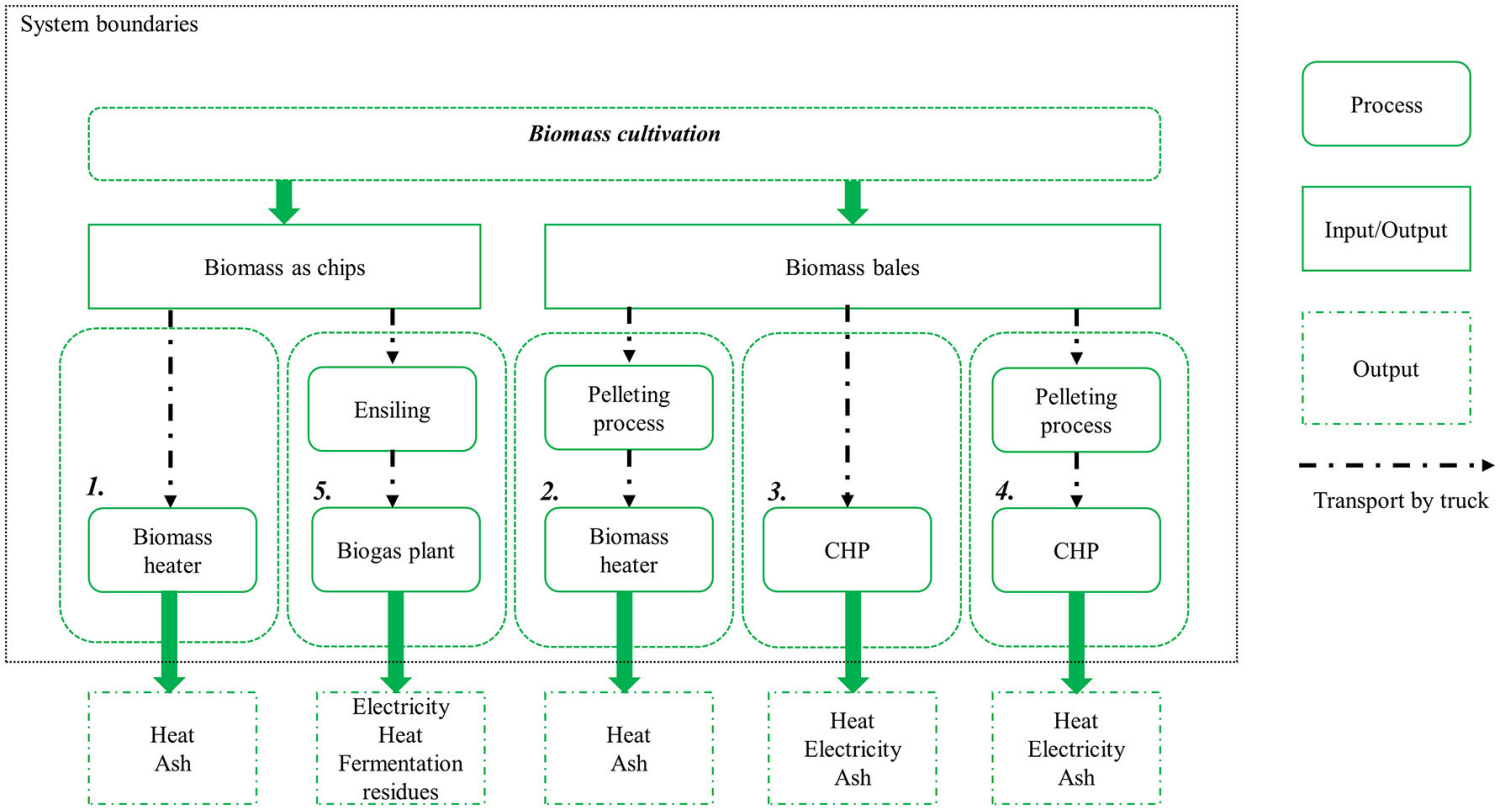
System description and boundaries for the energetic utilization pathways 1–5.

The utilization pathways 1 to 5 are shown in Figure 2. In all four combustion pathways (1, 2, 3, and 4), the handling of the ash is the same. It is assumed that both the fly and bottom ash is disposed of in landfill. The fly ash in particular has high levels of heavy metals. In utilization pathway 1, the miscanthus biomass is used on-farm in a small combustion unit to generate heat. In utilization pathway 2, miscanthus biomass in the form of pellets instead of chips is utilized in a small combustion unit to generate heat. The reference product of the utilization pathways 1 and 2 is heat produced by combustion of light fuel oil. This reference product was chosen, because it is produced in a comparable small-scale combustion unit. A sensitivity analysis was performed with heat produced by combustion of natural gas as a reference product to analyse the impact of this assumption.

In utilization pathway 3, miscanthus bales are combusted in a combined heat and power unit (CHP) to generate heat, with electricity as a co-product. In pathway 4, miscanthus pellets are utilized in the CHP instead of bales. Heat was specified as the main and electricity as the co-product in accordance with the description in the ecoinvent database (Weidema et al., 2013). The electricity produced is assumed to substitute the European electricity mix. The heat generated substitutes heat produced by the combustion of natural gas in a CHP. Natural gas was chosen in this case as a reference product, because it is a relative clean energy source (May and Brennan, 2006). This assumption reduces the risk of overestimating the net environmental benefit of the miscanthus-based alternative.

Utilization pathway 5 includes the fermentation of green-harvested miscanthus biomass to biogas and subsequent combustion to generate electricity, with heat as a co-product. Electricity was selected as main product in accordance with Bacenetti et al. (2016) and the European electricity mix was chosen as reference product. The heat generated as co-product substitutes heat produced by the combustion of natural gas in a CHP. The residues of the fermentation process are rich in nutrients (see Table S1) and can be used to substitute mineral fertilizer.

**Figure 3:**
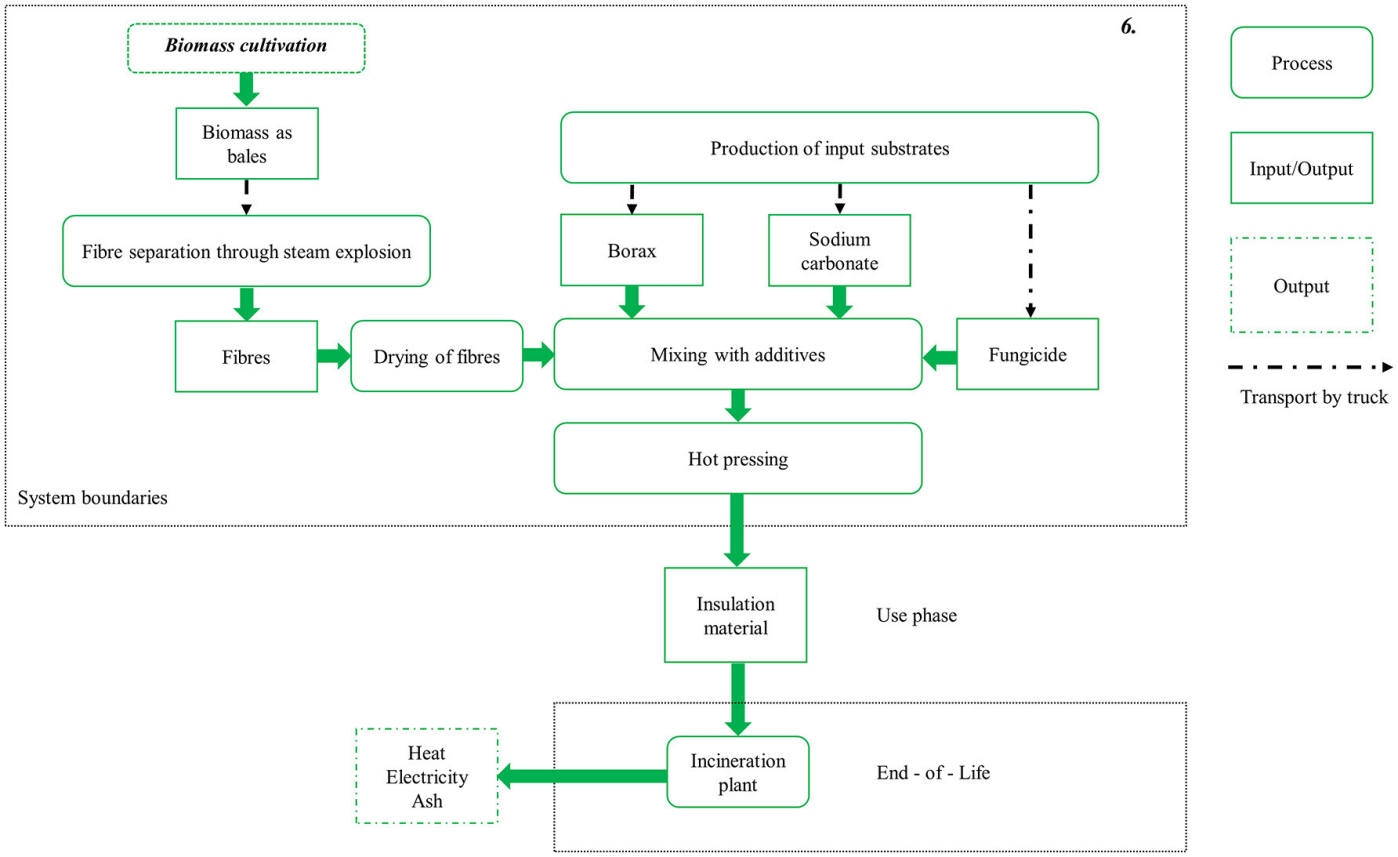
System description and boundaries for the material utilization pathway 6.

Utilization pathway 6, which is displayed in Figure 3, is the production of insulation material from miscanthus biomass. The miscanthus fibers are separated via steam explosion, dried, and mixed with additives. Insulation material is then produced through hot pressing. The reference product for 1 m^3^ miscanthus-based insulation material is 110 kg glass wool mats with comparable characteristics (Meyer et al., 2016). The End-of-Life of the miscanthus- and the fossil-based pathways are included in the assessment. The glass wool is treated as inert waste and disposed of to landfill. After its use phase, it is assumed that the miscanthus-based insulation material is incinerated, generating heat and electricity (see Figure 3). The electrical and thermal efficiencies of the incineration plant are comparable to the CHP plant used in the utilization pathways 3 and 4.

### Life cycle inventory

#### Agricultural system

The data used in this Life Cycle Assessment for the cultivation phase of miscanthus were obtained from multi-location field trials conducted within the OPTIMISC project (Lewandowski et al., 2016). Table 2 shows the main inputs and outputs at the different sites for the pathways using biomass harvested in spring (combustion, production of insulation material), or autumn (biogas substrate). Field data for pathway 5 was only available for the Adana, Moscow and Stuttgart sites (see Table 2).

**Table 2:** Summary of the main inputs and outputs of the spring and the autumn harvests.

**Values in kg yr^−1^ ha^−1^**	**Adana**	**Aberystwyth**	**Moscow**	**Potash**	**Stuttgart**	**Wageningen**
	**Harvest Feb./Mar**.					
N	60	60	60	60	60	60
K_2_O	120	120	120	120	120	120
P_2_O_5_	30	30	30	30	30	30
Herbicides	0.93	0.93	0.93	0.93	0.93	0.93
Dry matter yield	12,600	9,745	9,734	16,065	15,316	10,320
	**Harvest Sept./Oct**.					
N	140	n.a.	140	n.a.	140	n.a.
K_2_O	200	n.a.	200	n.a.	200	n.a.
P_2_O_5_	30	n.a.	30	n.a.	30	n.a.
Herbicides	0.93	n.a.	0.93	n.a.	0.93	n.a.
Dry matter yield	19,365	n.a.	15,568	n.a.	23,624	n.a.

In addition to the inputs shown in Table 2, the field trials in Adana were irrigated with 976.75 m^3^ water per hectare and year, independent of harvest date.

Nitrogen was applied as calcium ammonium nitrate, potassium as potassium sulfate and phosphate as triple superphosphate. Herbicides are only necessary in miscanthus cultivation in the preparation of the sites, in the first two cultivation years, when miscanthus is unable to compete with weeds, and in the recultivation process. Over the twenty-year cultivation period, a total application of 16.2 l herbicides ha^−1^ were applied: 10 l ha^−1^ Round up (Monsanto, active ingredient 360 g l^−1^ glyphosate); 3.5 l ha^−1^ Stomp Aqua (BASF, active ingredient 455 g l^−1^ pendimethalin); 1.5 l ha^−1^ Calisto (Syngenta, active ingredient 100 g l^−1^ mesotrione); 0.2 l ha^−1^ Arrat (BASF, active ingredient 100 g l^−1^ tritosulfuron and 500 g l^−1^ dicamba); and 1 l ha^−1^ Dash, (BASF, an emulsifiable concentrate). This corresponds to an average of 0.81 l or 0.93 kg ha^−1^ yr^−1^ herbicides.

The yield data in Table 2 is shown per year. However, these yield data are based on the whole cultivation period including the establishment phase. In the first year, the biomass is not harvested but mulched, and the full yield is only achieved from the third year onwards (Lewandowski et al., 2003). The calculation for the early spring harvest is given in Equation 1 and for the autumn harvest in Equation 2.

(1) $$\begin{array}{rc} Mean\;yield\;spring\;\left[t\;DM\;ha^{-1}yr^{-1}\right]\\ = & \frac{yield\;\left(2.\;year\_spring\;+\;3.year\_spring*18\right)}{20} \end{array}$$

(2) $$\begin{array}{rc} Mean\;yield\;autumn\;\left[t\;DM\;ha^{-1}yr^{-1}\right]\\ = & \frac{yield\;\left(2.\;year\_autumn\;+\;3.year\_autumn*18\right)}{20} \end{array}$$

Table 3 shows the agricultural operations applied during miscanthus cultivation including frequency. These are shown for two harvest procedures: in the chopping line, the biomass is processed to chips to be used in the utilization pathways 1 and 5; and in the baling line, it is baled (utilization pathways 2, 3, 4, and 6).

**Table 3:** Agricultural operations applied during 20 years of miscanthus cultivation with frequency.

**Agricultural operations**	**Frequency per cultivation period**	
	**Chopping line**	**Baling line**
Rotary harrow	2	2
Plowing	1	1
Planting	1	1
Mulching—first year	1	1
Spraying	5	5
Fertilizing	19	19
Mowing	0	18
Swath	0	18
Chipping	18	0
Baling	0	18
Mulching—final year	1	1
Chisel plow	1	1

The background data for the environmental impacts associated with the cultivation processes (e.g., plowing, mowing) and the production of the input substrates were taken from the ecoinvent database version 3.3 (cut-off system model) (Weidema et al., 2013). The energy demands of the harvesting processes (chopping and baling) and the pelleting process are based on Hastings et al. (under review).

N_2_O emissions from harvest residues and indirect N_2_O emissions from nitrogen fertilizer were estimated using emission factors based on IPCC (2006). Direct N_2_O and NO emissions from nitrogen fertilizer were calculated according to Bouwman et al. (2002). Ammonia emissions were calculated using emission factors from EMEP/CORINAIR (2001). Phosphate and phosphorus emissions to surface and groundwater, and heavy metal emissions to agricultural soil were estimated based on Nemecek and Kägi (2007). Nitrate leaching to groundwater was calculated according to the SQCB—NO_3_ model described in Faist Emmenegger et al. (2009). All pesticide applied have been modeled completely as emission to agricultural soil in accordance to Nemecek and Schnetzer (2011). The ecotoxicity values of this emission are based on the ecoinvent database (Weidema et al., 2013).

Several recent publication have demonstrated the ability of miscanthus to sequester CO_2_ in the soil through an increase in soil organic carbon, especially in comparison to annual plants (Gauder et al., 2016; McCalmont et al., 2017). However, these changes in soil organic carbon are highly dependent on the previous crop and thus contain a high degree of uncertainty (Harris et al., 2015). Because of this, carbon sequestration in the soil was not included this assessment.

Table 4 gives the farm-to-field distances and truck transport distances for the different utilization pathways. No data were available for the transport distances of input substrates (e.g., fertilizer) or propagation material. Therefore, a transport distance of 150 km for the input material by a EUR5 truck was assumed. The background data associated with the transportation of the input material and biomass were taken from the ecoinvent database (Weidema et al., 2013).

**Table 4:** Transport distances for the utilization pathways.

**Process**	**Unit**	**Utilization pathways**					
		**(1)**	**(2)**	**(3)**	**(4)**	**(5)**	**(6)**
Truck transport of input substrates	km	150	150	150	150	150	150
Farm-field distance	km	2	2	2	2	15	2
Truck transport of bales	km	–	100	400	100	–	400
Truck transport of pellets	km	–	400	–	400	–	–

There are considerable differences in transport density between chips, bales and pellets. To account for these differences, the emission data from the ecoinvent database used for the transport process (Weidema et al., 2013) was adapted in accordance with Hastings et al. (under review).

#### Utilization pathways

The following section describes the life cycle inventories for the different utilization pathways. The modeling of the pathways included the emissions associated with the construction of the conversion plants (e.g., CHP unit, biogas plant) and necessary infrastructure, based on background data from the ecoinvent database (Weidema et al., 2013).

The biomass heater used for utilization pathways 1 and 2 is a furnace with a heat generation capacity of 300 kW. The background data for the emissions associated with combustion is taken from the ecoinvent database. This data is based on a Froling Turbomat 320 kW woodchip boiler with a thermal efficiency of 75%. This is lower than in the technical specification, because it represents the average annual operation, which includes start and stop phases (Weidema et al., 2013).

The background emission data for utilization pathways 3 and 4 [combined heat and power unit (CHP)] are based on the ecoinvent process “*heat and power co-generation, wood chips, 6,667 kW, state-of-the-art 2014.”* According to the process description in the ecoinvent database, an organic rankine cycle (ORC) steam generator with an electrical efficiency of 15% and a thermal efficiency of 45% is used (Weidema et al., 2013).

As there is insufficient specific information available on emissions from miscanthus combustion, all four utilization pathways are based on wood combustion processes. Miscanthus-specific emission factors for carbon monoxide, sulfur dioxide, hydrogen chloride, nitrogen oxides, and particulates were taken from Dahl and Obernberger (2004). At the time of harvest, miscanthus biomass has a water content of around 15% (Lewandowski et al., 2016). A further drying process is therefore not necessary. A mean calorific value of 4.3 kWh kg^−1^ fresh biomass was calculated based on the model of Jiménez and González (1991).

The miscanthus biomass used in the biogas plant is harvested in autumn and then ensiled. Dry matter losses of 12% were assumed during the ensilage process. The silage is subsequently fermented to biogas. The methane hectare yield [m^3^ CH_4_ yr^−1^ ha^−1^] for the Adana site was 4,676, for the Moscow site 4,194, and for the Stuttgart site 6,495 (Kiesel et al., 2017). The methane yield was measured as described in Kiesel and Lewandowski (2017). A biogas batch test was performed for 35 days in mesophilic conditions (39°C) according to VDI guideline 4,630. The approach of the biogas batch test was certified by the KTBL and VDLUFA interlaboratory comparison test 2014 and 2015. Each sample was assessed in four technical replicates. Methane losses of 1% were assumed in the biogas plant based on Börjesson and Berglund (2007). The biogas is combusted in a CHP unit to generate heat and power. The electricity is fed into the grid. Twenty percent of the heat produced is used internally for the heating of the fermenter. In this study, it was assumed that 50% of the remaining heat (that is 40% of the total heat produced) is used to heat nearby residential buildings and so substitute heat produced from fossil sources. The other 50% of the remaining heat is not used and thus is excess heat that escapes into the atmosphere. The technical characteristics of the CHP used in this study are shown in Table 5 (Uihlein et al., 2008). Both the emissions associated with biogas combustion in the CHP unit and the construction of the biogas plant are based on the ecoinvent database (Weidema et al., 2013).

**Table 5:** Technical characteristics of the biogas plant used in the analysis.

**Technical characteristics**		**Unit**
Full load hours	7,800	H
Plant output electrical	500	kWh_el_
Plant output total	1,351	kWh
Electrical efficiency	37	% of plant total output
Thermal efficiency	53	% of plant total output
Inherent heat demand	20	% of total heat production
Inherent power consumption	12	% of total power production

To produce 1 m^3^ of insulation material, 194.3 kg dry-matter miscanthus biomass is required. This corresponds to 228.6 kg fresh biomass at a moisture content of 15%. The additives consist of 3.85 kg borax, 3.85 kg sodium carbonate and 1.1 kg of the fungicide thiocarbamate (Velásquez et al., 2003). The energy required for the production process is shown in Table 6.

**Table 6:** Energy consumption for the production of miscanthus-based insulation material.

**Energy consumption**	**Unit**	**Per kg dry-matter miscanthus biomass**	**Per m^3^ insulation material**
Steam explosion	MJ_th_	1.452	282.085
	MJ_el_	0.073	14.104
Drying of fibers	MJ_th_	1.493	290.111
	MJ_el_	0.075	14.506
Mixing and hot pressing	MJ_th_	0.824	160.103
	MJ_el_	0.042	8.161
Total	MJ_th_	3.769	732.299
	Mj_el_	0.19	36.771

### Choice of impact categories

The life cycle impact assessment methodology ReCiPe was used in this LCA study (Goedkoop et al., 2008). All 18 mid-point indicators described in this methodology were included: climate change (CC), which corresponds to global warming potential (GWP); ozone depletion (OD); terrestrial acidification (TA); freshwater eutrophication (FE); marine eutrophication (ME); human toxicity (HT); photochemical oxidant formation (POF); particulate matter formation (PMF); terrestrial ecotoxicity (TET); freshwater ecotoxicity (FET); marine ecotoxicity (MET); ionizing radiation (IR); agricultural land occupation (ALO); urban land occupation (ULO); natural land transformation (NLT); mineral resource depletion (MRD); fossil fuel depletion (FFD); and water depletion (WD). The results are shown as normalized values. This means, that the results of each impact category are divided by the respective emissions caused by an average European in the year 2000. The resulting values show the calculated impact as a proportion of the emissions of an average European citizen. The characterization and normalization factors are based on Goedkoop et al. (2008). No normalized values are given for the impact category “water depletion,” as no normalization factor is available in the ReCiPe methodology for this impact category (Goedkoop et al., 2008).

## Results

**Figure 4:**
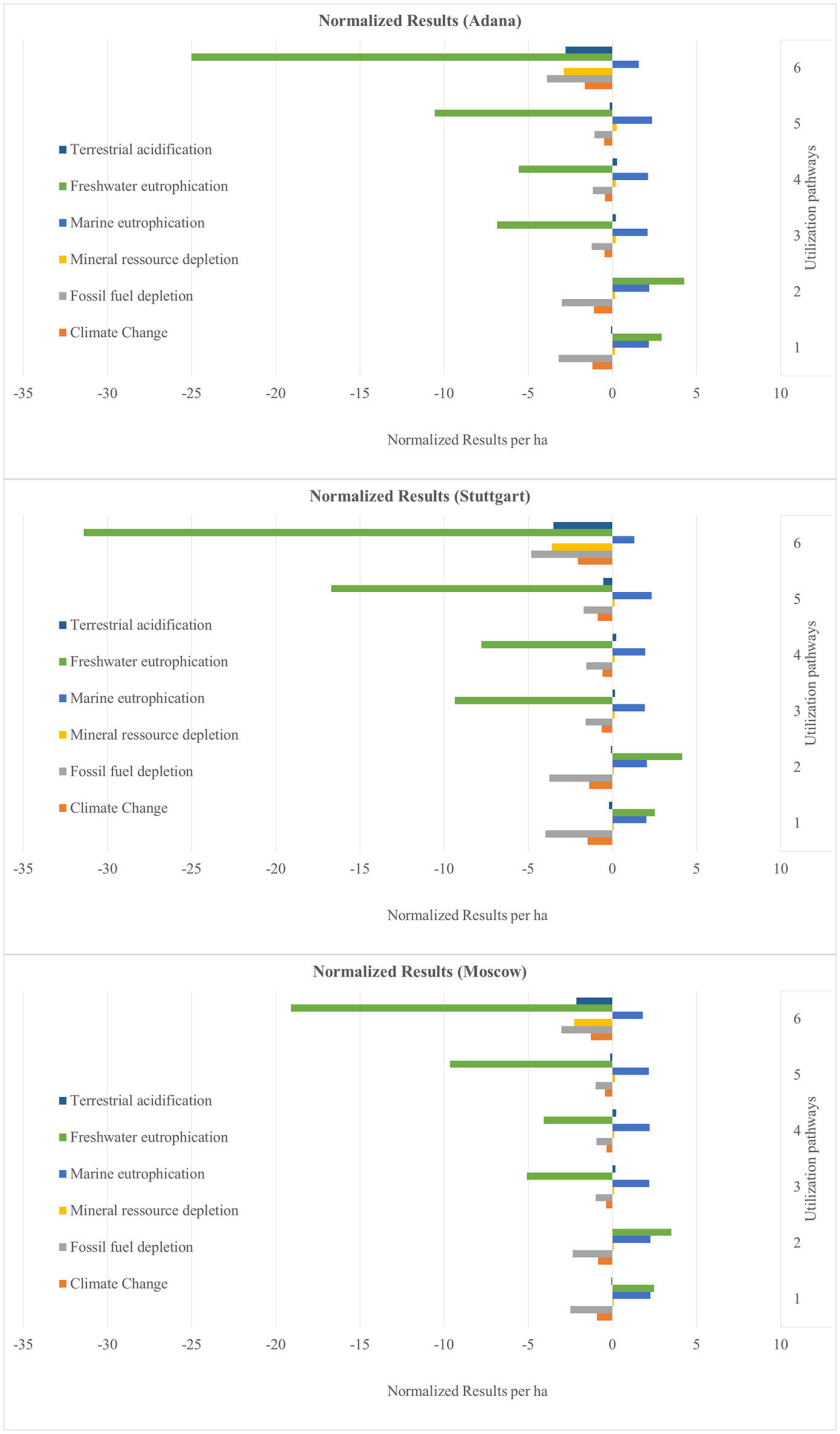
Normalized results per ha for the sites Adana, Stuttgart, and Moscow—Part 1. Utilization pathways: 1. Small-scale combustion—chips; 2. Small-scale combustion—pellets; 3. Large-scale combustion—biomass baled for transport and storage; 4. Large-scale combustion—pellets; 5. Medium-scale biogas plant—biomass ensiled; and 6. Large-scale production of insulation material—biomass baled for transport and storage.

**Figure 5:**
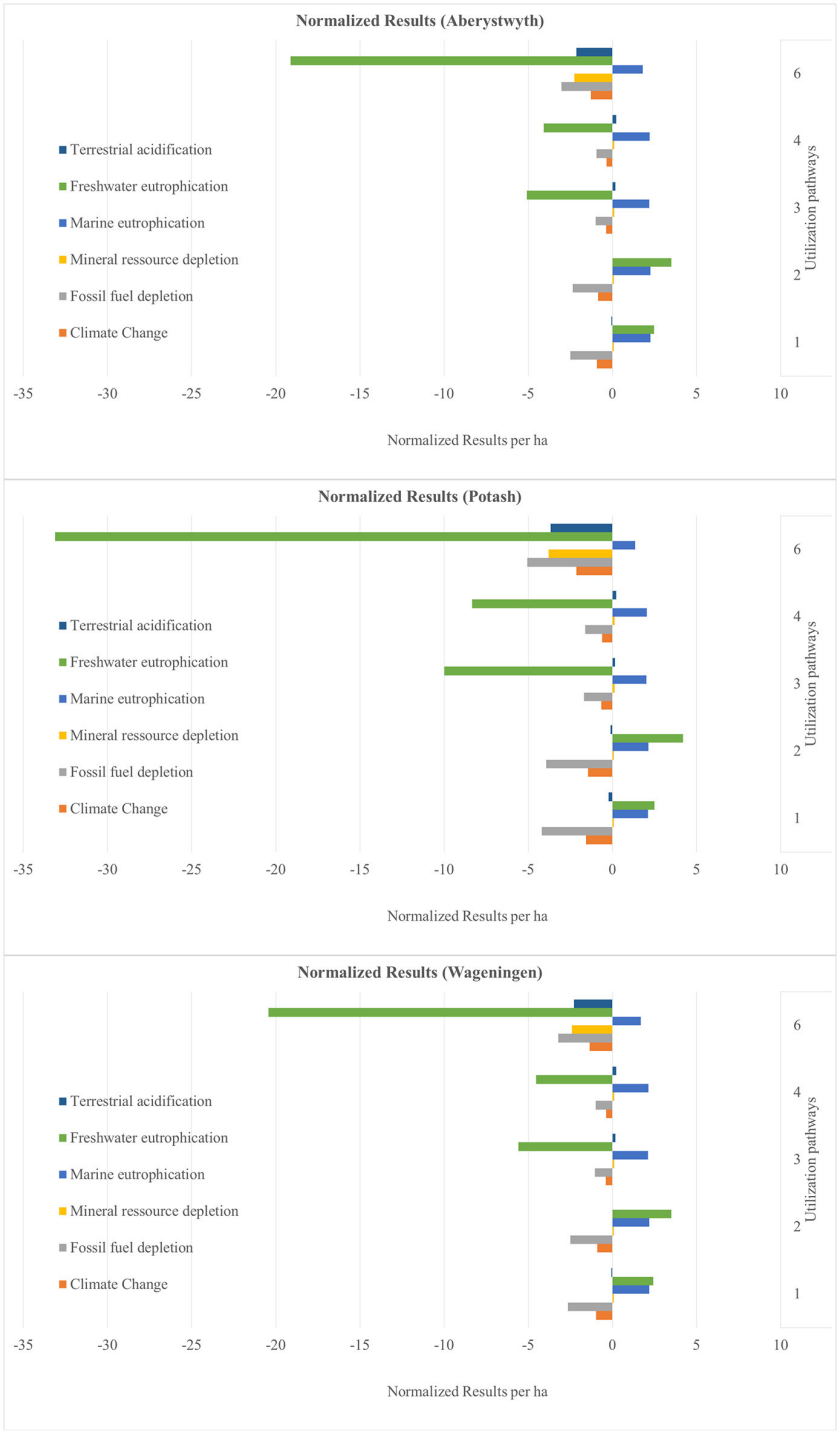
Normalized results per ha for the sites Aberystwyth, Potash, and Wageningen–Part 1. Utilization pathways: 1. Small-scale combustion—chips; 2. Small-scale combustion—pellets; 3. Large-scale combustion—biomass baled for transport and storage; 4. Large-scale combustion—pellets; and 6. Large-scale production of insulation material—biomass baled for transport and storage.

**Figure 6:**
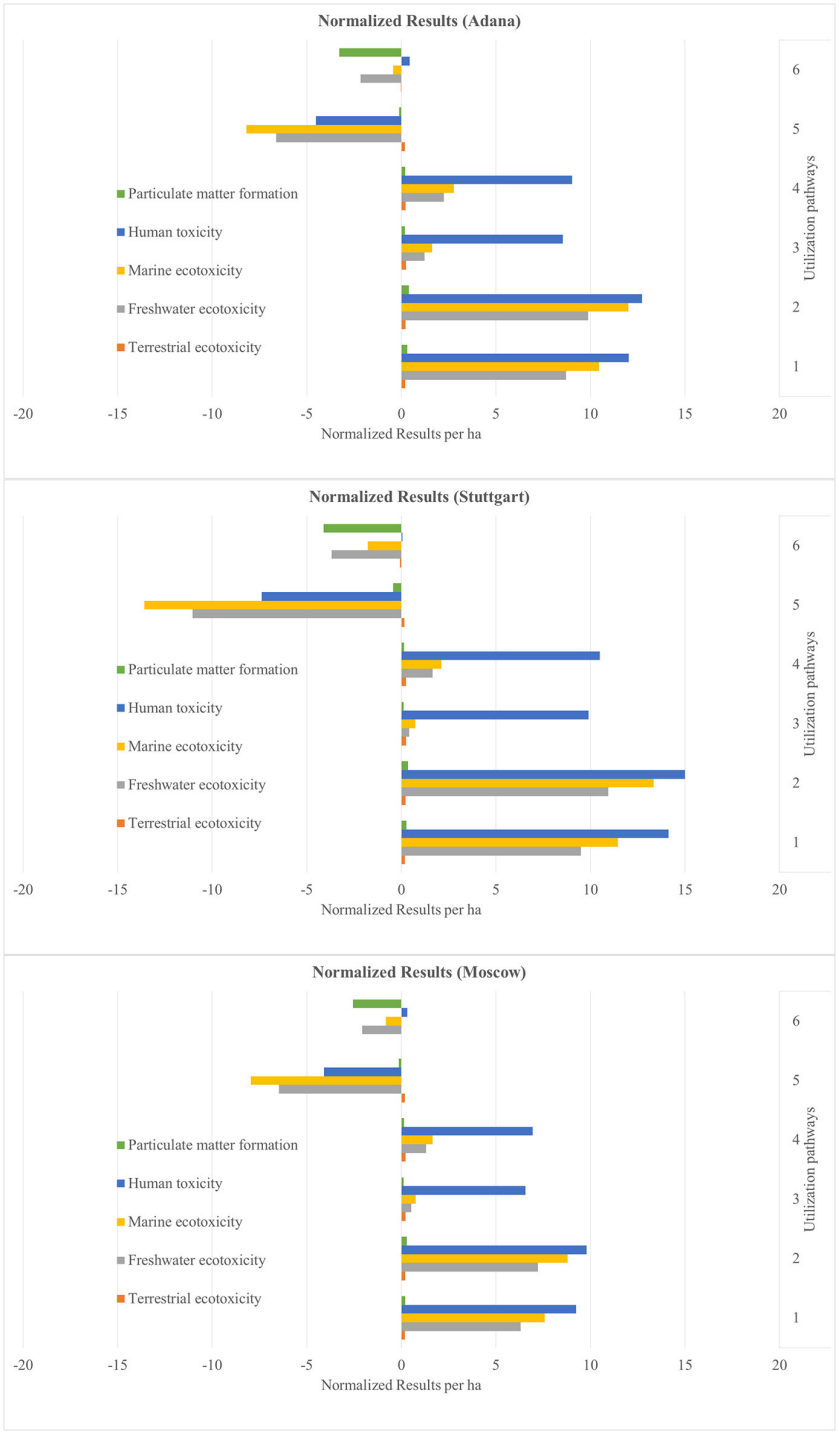
Normalized results per ha for the sites Adana, Stuttgart, and Moscow—Part 2. Utilization pathways: 1. Small-scale combustion—chips; 2. Small-scale combustion—pellets; 3. Large-scale combustion—biomass baled for transport and storage; 4. Large-scale combustion—pellets; 5. Medium-scale biogas plant—biomass ensiled; and 6. Large-scale production of insulation material—biomass baled for transport and storage.

**Figure 7:**
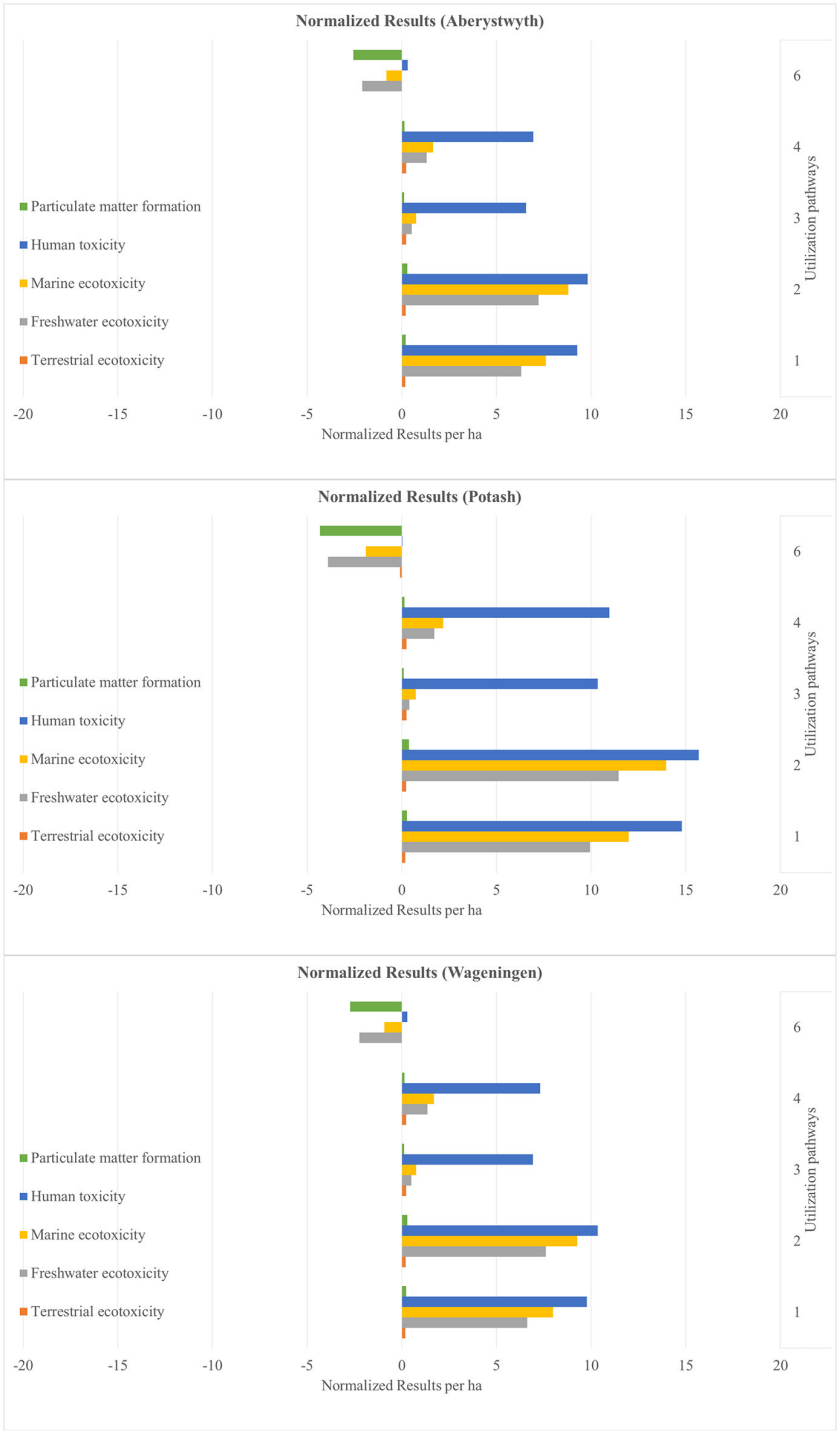
Normalized results per ha for the sites Aberystwyth, Potash, and Wageningen—Part 2. Utilization pathways: 1. Small-scale combustion—chips; 2. Small-scale combustion—pellets; 3. Large-scale combustion—biomass baled for transport and storage; 4. Large-scale combustion—pellets; and 6. Large-scale production of insulation material—biomass baled for transport and storage.

**Figure 8:**
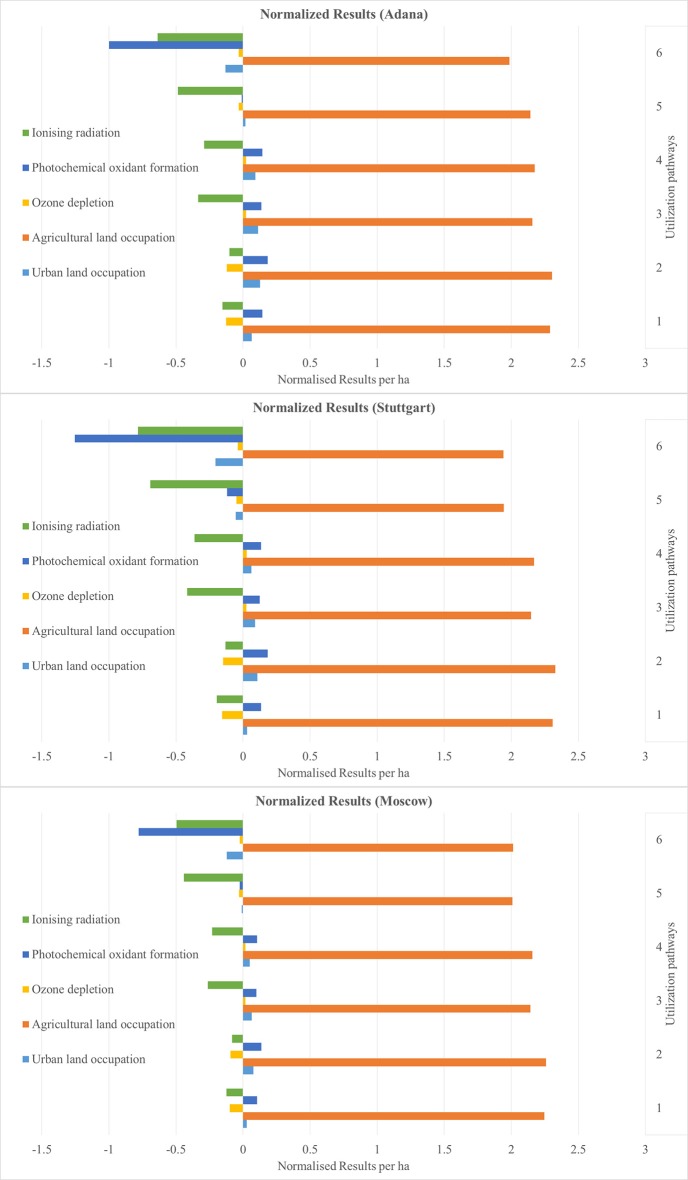
Normalized results per ha for the sites Adana, Stuttgart, and Moscow—Part 3. Utilization pathways: 1. Small-scale combustion—chips; 2. Small-scale combustion—pellets; 3. Large-scale combustion—biomass baled for transport and storage; 4. Large-scale combustion—pellets; 5. Medium-scale biogas plant—biomass ensiled; and 6. Large-scale production of insulation material—biomass baled for transport and storage.

**Figure 9:**
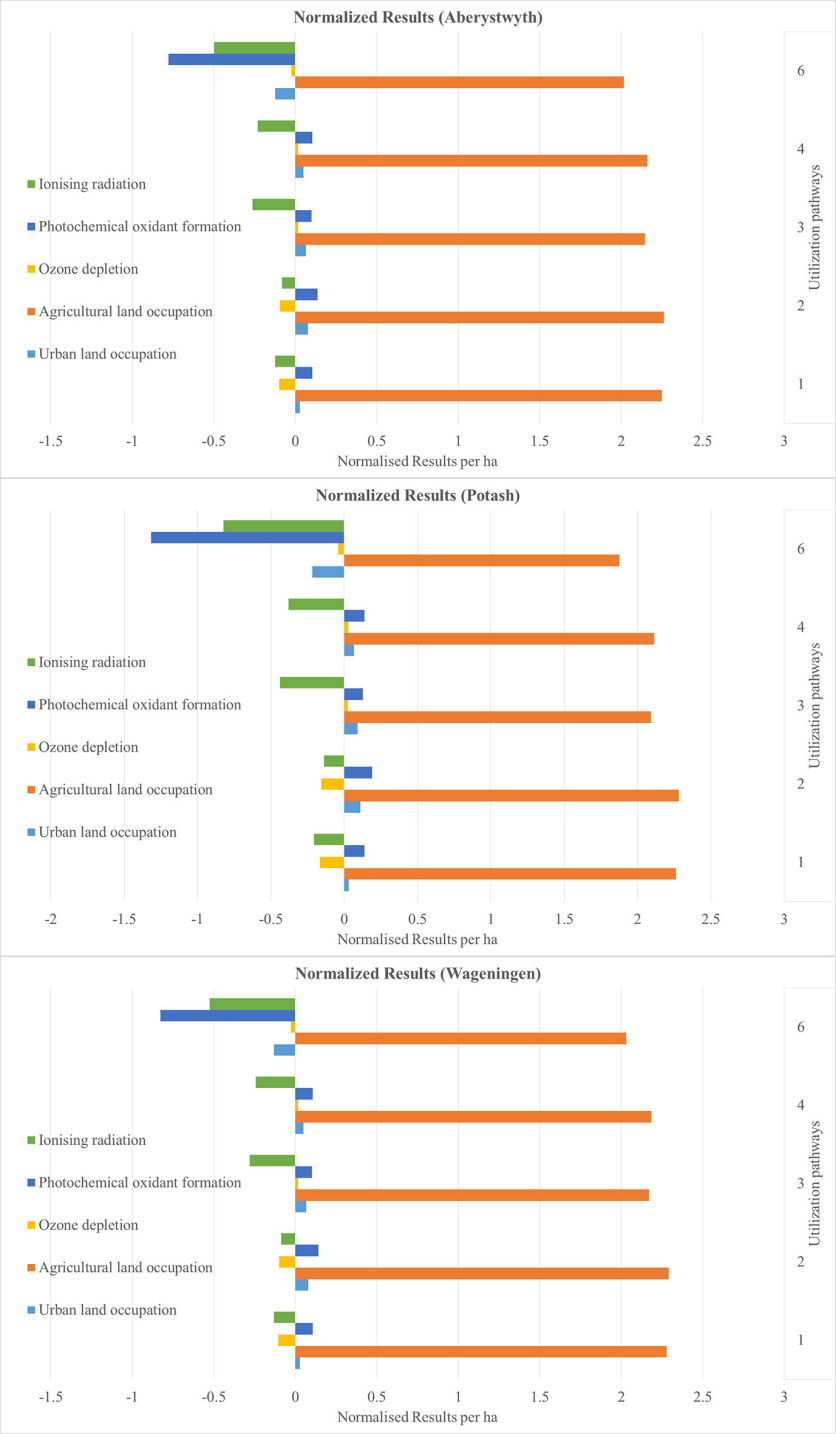
Normalized results per ha for the sites Aberystwyth, Potash, and Wageningen—Part 3. Utilization pathways: 1. Small-scale combustion—chips; 2. Small-scale combustion—pellets; 3. Large-scale combustion—biomass baled for transport and storage; 4. Large-scale combustion—pellets; and 6. Large-scale production of insulation material—biomass baled for transport and storage.

The results are presented as normalized values. These show the net benefits and impacts of the utilization of 1 ha miscanthus for all six sites and for all six utilization pathways (see Figures 4–**9**). The absolute values per ha for all utilization pathways on all sites analyzed are given in the Supplementary Material (Tables S2–S7). In addition, they are shown per MJ_th_ for the utilization pathways 1, 2, 3, and 4 (Tables S2–S5), in MJ_el_ for utilization pathway 5 (Table S6) and in m^3^ insulation material for utilization pathway 6 (Table S7).

The normalized net benefits and impacts per ha in the impact categories TA, FE, and ME, MRD and FFD, and CC are shown in Figure 4 for the sites Adana, Stuttgart and Moscow and in Figure 5 for the sites Aberystwyth, Potash, and Wageningen. Utilization pathway 6 (production of insulation material) has the largest net benefits in the categories TA, FE, MRD, and CC on all sites. This is due to the substitution of the reference product glass wool, which has a very emission-intensive production process. All utilization pathways perform negatively in the category ME. This is largely caused by nitrogen-fertilizer-induced nitrate emissions in the miscanthus cultivation process. Utilization pathways 1 and 2 (both small-scale combustion) also have a negative impact in FE, which is mainly caused by phosphate-fertilizer-induced emissions. The production process of the reference product of utilization pathways 1 and 2 (heat generated through the combustion of light fuel oil) has a low FE. For this reason, the substitution caused a net negative impact on the environment in this category. Differences between the utilization pathways 1 and 2, as well as 3 and 4 are due to differences in transport distance and the additional pelleting process. As a result, pathway 1 has lower environmental impacts than pathway 2, and pathway 3 lower environmental impacts than pathway 4. This applies to all impact categories.

The normalized net benefits and impacts per ha in the impact categories PMF, HT, MET, FET, and TET for the sites Adana, Stuttgart and Moscow are shown in Figure 6, and for the sites Aberystwyth, Potash and Wageningen in Figure 7. The utilization pathway 5 (medium-scale biogas plant) had relatively high environmental benefits in HT, MET, and FET (see Figure 6). These can be explained by the emission-intensive production process of the substituted reference product, the European electricity mix. Utilization pathway 6 showed low environmental impacts in the category PMF compared with the other utilization options. This is due to the high impact of the substituted reference product glass wool in this impact category, in particular its production process. All other utilization pathways had a comparatively negative performance in all impact categories depicted in Figures 6, 7. The net impacts in ME, FE and especially HT in the utilization pathways 1 to 4 result from the treatment of the bottom and fly ash, which incur in the combustion process.

The normalized net benefits and impacts per ha in the impact categories IR, POF, OD, ALO, and ULO are shown in Figure 8 for the sites Adana, Stuttgart, and Moscow, and in Figure 9 for the sites Aberystwyth, Potash, and Wageningen. Naturally, all biomass-based utilization pathways perform negatively in the category ALO. Utilization pathway 6 shows a comparatively large net benefit in the category POF. This is again caused by the substitution of the reference product. The net benefit of utilization pathways 1 and 2 in the category OD result from the emission-intensive generation of the reference product (heat generated by the combustion of light fuel oil). All utilization pathways had a comparatively large net benefit in the impact category natural land transformation (data not shown). The normalized results range from −6.15 for utilization pathway 5, to −42.86 for utilization pathway 1. In all utilization pathways, this is caused by the substituted reference products, which have a strong negative impact in this category. For clarity of presentation, these results are not included in Figures 8, 9 due to their considerably higher values.

## Discussion

The first part of the discussion focuses on the normalized values shown in Figures 4–9, including a critical reflection on the influence on the final results of reference product selection and credits given for co-products. In addition, the impact of the End-of-Life phase of the products is elaborated. The second part discusses the relevance of the impact categories for the various utilization pathways analyzed in this study. The final part gives recommendations for improving the environmental performance of the biobased value chains and considers the implications of the results for future biomass use.

### Determinants of environmental benefits and impacts

Figures 4–9 show the normalized values for the environmental benefits and impacts per hectare (including the cultivation of the biomass and subsequent utilization) minus the substitution of a reference product and the credits given for co-products.

A comparison of the normalized results from this study with results from reference literature is only partially possible due to different assumptions, system boundaries and methodologies used. Wagner and Lewandowski (2017) analyzed the relevance of various impact categories for a small-scale combustion chain using miscanthus and willow cultivated under three nitrogen fertilizer regimes. The results of their study show strong similarities with those of the current assessment, in particular with regard to the question of which impact categories are relevant and which not.

In general, the utilization pathways 5 (fermentation of miscanthus in a biogas plant and subsequent utilization in a CHP) and 6 (production of insulation material) had the lowest impacts on the environment. They had considerably larger net benefits, especially in the impact categories MET and FET, and FE. The results of the small-scale combustion chains again emphasized the necessity of including more impact categories than just climate change when analyzing and comparing the environmental performance of biobased utilization pathways (Jeswani et al., 2015; Wagner and Lewandowski, 2017). The small-scale combustion chains had advantages in the impact categories OD and FFD, and achieved the highest climate change saving potential of all energetic value chains (1, 2, 3, 4, and 5). However, they scored worse in most of the other impact categories. This also emphasizes the difficulty of determining the most sustainable utilization option from an environmental point of view. One way of resolving this issue is to combine the results of several impact categories into a single score for the total environmental sustainability (Rajagopalan et al., 2017). However, such an aggregation reduces the overall transparency of the results (Bare et al., 2000).

There is a large variation in the results between the six sites and between the six utilization pathways. The site differences are chiefly caused by variations in yield. The differences between the utilization pathways have several causes: the reference products have the largest impact, but the credits given for co-products and the effect of End-of-Life phase also play an important role. These four factors with a strong influence on the environmental benefits and impacts are discussed in the following sub-sections.

#### Influence of the variability of the biomass yield

The average yields used in this assessment are based on the yield measured in the third year and are at the lower end of those of other studies (Christian et al., 2008; Iqbal et al., 2015). In this study, it was assumed that full yields are reached from the third year onwards. However, other studies analyzing long-term field trials suggest that full yields are only achieved from the fourth year onwards (Christian et al., 2008; Iqbal et al., 2015). That would mean that the yields used in this study are conservative assumptions and could be higher over the whole cultivation period.

The differences between the six sites for the same utilization pathways seen in Figures 4–9 can be attributed to differences in yield. Sites on which significantly higher yields were achieved (e.g., Potash and Stuttgart) showed a better environmental performance. Other studies also emphasize the importance of yield for environmental performance (Meyer et al., 2016). However, it is worth mentioning that the influence of yield variation only changed an impact into a benefit, or vice versa, in very few impact categories, independent of utilization pathway (see Figures 4–9). Aberystwyth was a particularly interesting site; the values for the environmental benefits here were low compared to the other sites. The reason for that is that, in Aberystwyth, the yield was lower because the miscanthus was grown on marginal land. However, some utilization pathways, such as production of insulation material, still achieved comparatively low impacts on the environment even though the miscanthus was cultivated under marginal conditions.

#### Influence of the selection of the reference product

The selection of an appropriate reference product is essential for the accuracy of the assessment, especially in the case of the heat-producing value chains 1–4 (Wolf et al., 2016). For the utilization pathways 1 and 2 (small-scale combustion), heat produced by combustion of light fuel oil was substituted. Changing the reference product to natural gas alters the results substantially. The net impact for the category MRD increases by 231%, for PMF by 220%, and for POF by 220%. In addition, the climate change saving potential is reduced by 77% and the benefit in the impact category fossil fuel depletion is reduced by 66%. This sensitivity analysis clearly shows the influence of the selection of the reference product on the result of the assessment. Furthermore, it emphasizes how crucial it is in practice to first phase out emission-intensive power plants based on coal and fuel oil, rather than those based on natural gas. However, the change of the reference product in utilization pathways 1 and 2 only turns a net benefit into an impact in the impact categories ionizing radiation and terrestrial acidification. The results of this sensitivity analysis are shown in the Supplementary Material (Table S8).

Heat generated by the combustion of natural gas was selected as reference product for the utilization pathways 3 and 4. Natural gas is a fossil energy carrier with comparatively low environmental impacts (May and Brennan, 2006), thus reducing the risk of overestimating the benefits of substitution by miscanthus-based heat. However, this also means that the environmental performance of the utilization pathways 3 and 4 can be improved considerably if heat generated by the combustion of fuel oil or coal is substituted.

The European electricity mix was used as reference product for the energetic utilization pathway 5. The choice of this reference is one reason for the low impacts on the environment of this utilization pathway. As electricity is an energy form with higher emissions per MJ than heat generation, the net benefits of its substitution are also higher. It should be noted that in this study an electricity mix was used as a reference product, which also includes electricity from renewable sources (Weidema et al., 2013). If only electricity generated by fossil sources is substituted, the environmental performance can be further improved.

#### Influence of credits given for co-products

For those utilization pathways with more than one product, credits were given for the co-products. This was the case for the electricity produced as co-product in the CHP unit in the utilization pathways 3 and 4. The CHP produced 0.3 MJ of electricity for every MJ heat and it was assumed that this electricity substituted a European electricity mix. As already mentioned above, electricity has higher negative impacts on the environment than heat. That is why, in most impact categories, the credits given for the co-product were higher than the effect of substituting the reference product (see Table S9). The utilization pathway 5 produces heat as a co-product, which is partly utilized to heat nearby buildings, thus substituting fossil-based heat. In addition, the fermentation residues are rich in nutrients and can be used to substitute mineral fertilizers. These residues are a particularly valuable resource and the credits given for their utilization improve the environmental performance of this pathway considerably. The values used for these credits are displayed in Table S10.

#### Influence of the inclusion of the end-of-life phase

The inclusion of the End-of-Life of biobased products is also an important point with a strong influence on their environmental performance. The insulation material produced in pathway 6 is first used as a biobased construction material and after the use phase incinerated in a CHP. The positive influence of this multiple use is important for the relatively low impacts on the environment of miscanthus-based insulation material. For example, the production of this insulation material (including the cultivation phase on the Stuttgart site and the truck transport of the biomass) causes around 124 kg CO_2_ eq. per m^3^. Of this, around 117 kg CO_2_ eq. can be recovered through its incineration, generating heat and power which substitute conventionally produced energy. In the impact category terrestrial acidification, 0.58 kg SO_2_ eq. per m^3^ are saved through this energy recovery, which is more than are emitted in the whole value-chain including the production process (0.42 kg SO_2_ eq.). These advantages of multiple use in comparison to single use have also been shown in other studies (Höglmeier et al., 2014, 2015). Another advantage of material use is the temporal storage of carbon in the product (Sikkema et al., 2013). This storage function can help decelerate climate change.

### Relevance of different impact categories

The normalization step applied enables the assessment of the relevance of the different impact categories for the environmental performance of each utilization pathway (Wagner and Lewandowski, 2017). There are large variations in relevance within the utilization pathways and within the impact categories analyzed. Once the relevance of an impact category has been established, it becomes evident which need to be included in a holistic analysis of the environmental performance of miscanthus-based value chains. The relevance of the impact categories should not only be evaluated in general but also for each specific utilization pathway. This knowledge assists the selection of the impact categories that require further improvement in each pathway.

The following section classifies the impact categories according to their normalized values into three groups: impact categories of (1) low relevance; (2) average relevance and (3) high relevance.

Several impact categories have comparatively low normalized impacts or benefits on the environment in most pathways and are therefore deemed of low relevance. These include: terrestrial acidification (TA), mineral resource depletion (MRD), particulate matter formation (PMF), ionizing radiation (IR), ozone depletion (OD), urban land occupation (ULO), photochemical oxidant formation (POF), and terrestrial ecotoxicity (TET). In addition, as the model and the LCI data used contain some uncertainties, small differences of ± 2 in normalized values are not considered significantly different.

The impact categories marine eutrophication (ME) and fossil fuel depletion (FFD) are deemed of average relevance. They should be included in the assessment, if the utilization pathways analyzed are expected to have a substantial impact in these categories. This is the case for ME, when higher amounts of nitrogen fertilizer are applied. The ME then increases considerably because higher nitrogen fertilizer application leads to an increase in nitrate leaching, the main cause of ME. As the production process of mineral nitrogen fertilizer is quite energy-intensive, FFD should also be included, when higher amounts of nitrogen fertilizer are applied. The FFD should also be assessed if the production phase of the utilization pathways analyzed requires large amounts of energy.

On the basis of the comparatively high normalized results, the impact categories human toxicity (HT), marine (MET), and freshwater ecotoxicity (FET) and freshwater eutrophication (FE) are considered very relevant for the assessment of miscanthus-based value chains. These results usually represent a substantial net impact for the combustion chains and a considerable net benefit for utilization in a biogas plant and production of insulation material.

The impact categories climate change (CC) and agricultural land occupation (ALO) are both deemed of high relevance, even if they have comparably low normalized impacts or benefits. This is due to the related environmental and social problems, which are of high interest to society in general. Climate change, for example, is presently one of the most urgent environmental problems and, as a result, this impact category is included in virtually every study which assesses the environmental performance of miscanthus-based value chains (Godard et al., 2013; Parajuli et al., 2015; Roy et al., 2015). The ALO can be a problem if the utilization of land for biomass production leads to land-use competition and thus hinders the production of food crops.

Although the normalization of the results allows the evaluation of the relevance of different impact categories, this method has its limitations. For example, it does not consider social preferences. In addition, the preload of the environment is not taken into account. For this reason, the results of the relevance assessment always need to be adapted according to the goal and scope of the respective study.

### How to improve the environmental performance

The relevance of the different impact categories also helps to identify potential for improvement by starting the focus on the categories with the highest normalized scores. The high values of the combustion chains for HT are caused by the treatment of the ash, which is rich in heavy metals. In this study the entire ash was disposed of to sanitary landfill. A separation into fly ash and coarse ash could improve the environmental performance. In this case, only the fly ash, which contains most of the heavy metals, would be disposed of to landfill and the coarse ash, which is rich in phosphate and potassium, could be used as fertilizer (Pitman, 2006). Performance in MET and FET is also problematic, especially for the combustion chains. The combustion process of the miscanthus biomass is responsible for the largest share of the emissions in these impact categories. Improvements in the emission control systems of the combustion unit would be one possibility to decrease the impacts in these categories. Another could be adaption of the harvest date and selection of the genotype in order to utilize biomass that contains less elements which lead to harmful emissions in the combustion process (Iqbal and Lewandowski, 2014).

The impact category ALO chiefly describes the area of agricultural land needed to produce the amount of biomass required for each utilization pathway. If it is possible to obtain higher yields per hectare, less land would be needed to produce the same amount of biomass and thus the ALO would decrease. Another possibility would be to increase the use efficiency of the biomass utilization pathways, so that less biomass is needed to produce the same amount of products.

The ME is mostly caused by nitrate leaching through the use of nitrogen fertilizers. Nitrogen-fertilizer-induced emissions in form of N_2_O are also a main hot spot in the impact category CC. Thus, a decrease in the amount of nitrogen fertilizer used would decrease the impact in these categories. Another possibility for improvement would be the use of nitrification inhibitors (Akiyama et al., 2010). In the impact category FFD, there is a clear distinction between the energetic (1, 2, 3, 4, 5) and the material (6) utilization pathways. The hot spots in the energetic pathways are the harvest, biomass transport to the conversion plant and pelleting process (where applicable). In utilization pathway 6 (insulation material), the production process is the main hot spot and has the largest potential for improvement, for example, through the use of renewable instead of fossil-based energy forms.

### Outlook

The utilization pathways modeled in this assessment are all based on novel genotypes, except at the Adana site. These novel genotypes were more suitable than the standard genotype *Miscanthus* × *giganteus* for the utilization pathways analyzed, based on yield and quality parameters (Lewandowski et al., 2016). Thus, the environmental performance assessed in this study reflects the advances made in recent years in both agricultural management and miscanthus breeding. The results reveal substantial differences in environmental performance between the various utilization pathways. Furthermore, they emphasize the advantages of the multiple use of biomass (as in the case of insulation material) compared to single use as an energy carrier. In order to increase the environmental benefits of biomass-based value chains, in future the material use of biomass should be favored.

Another relevant outcome of this study was the demonstration of the positive environmental performance of marginal land for miscanthus biomass production and utilization. In a developing European bioeconomy with a steadily increasing demand for biomass, this is a promising opportunity to boost biomass production without competing with food crops.

## Author contributions

MW was performing the LCA modeling and was leading the writing process. AK, AH, and YI provided data and contributed to the material and method parts and thus supporting the creation of the Life cycle inventory. Furthermore AH supported the modeling process and AK the discussion of the results. IL added valuable contribution to each chapter and in manifold discussions.

### Conflict of interest statement

The authors declare that the research was conducted in the absence of any commercial or financial relationships that could be construed as a potential conflict of interest.
