# Supplementary material for: Novel Miscanthus Germplasm-Based Value Chains: A Life Cycle Assessment
Source: Front Plant Sci. 2017 Jun 8;8:990. doi: 10.3389/fpls.2017.00990 (PMC5462955; doi:10.3389/fpls.2017.00990)
Supplement: Supplementary file 1 [file Table1.DOCX]

Table S1: Equivalent nutrients that can be displaced by the use of fermentation residues in utilization pathway 5

| **Location** | **N** | **K_2_O** | **P_2_O_5_** |
| --- | --- | --- | --- |
|  | **in kg/MJ_el_** | | |
| Adana | 0.00120 | 0.00311 | 0.00085 |
| Moscow | 0.00100 | 0.00261 | 0.00064 |
| Stuttgart | 0.00194 | 0.00172 | 0.00060 |
