# Supplementary material for: Novel Miscanthus Germplasm-Based Value Chains: A Life Cycle Assessment
Source: Front Plant Sci. 2017 Jun 8;8:990. doi: 10.3389/fpls.2017.00990 (PMC5462955; doi:10.3389/fpls.2017.00990)
Supplement: Supplementary file 2 [file Table2.DOCX]

Table S2: Environmental benefits and impacts per ha and MJ_th_ for utilization pathway 1 [Small-scale combustion – chips]

| **Results LCIA** | **Reference unit** | **Locations [results per ha]** | | | | | |
| --- | --- | --- | --- | --- | --- | --- | --- |
|  |  | **Adana** | **Aberystwyth** | **Moscow** | **Potash** | **Stuttgart** | **Wageningen** |
| Agricultural land occupation | m^2^*a | 10214.07 | 10119.50 | 10119.46 | 10142.86 | 10140.09 | 10121.63 |
| Climate Change | kg CO_2_ eq. | -13085.39 | -10167.31 | -10155.82 | -17366.78 | -16515.00 | -10825.14 |
| Fossil fuel depletion | kg oil eq. | -4954.23 | -3879.90 | -3875.24 | -6503.24 | -6192.41 | -4118.54 |
| Freshwater ecotoxicity | kg 1,4-DB eq. | 95.78 | 69.41 | 69.34 | 109.36 | 104.63 | 73.05 |
| Freshwater eutrophication | kg P eq. | 1.24 | 1.03 | 1.03 | 1.07 | 1.06 | 1.03 |
| Human toxicity | kg 1,4-DB eq. | 7537.77 | 5806.18 | 5800.02 | 9269.50 | 8859.15 | 6121.23 |
| Ionising radiation | kg U235 eq. | -957.43 | -768.27 | -767.35 | -1284.31 | -1223.17 | -815.21 |
| Marine ecotoxicity | kg 1,4-DB eq. | 91.31 | 66.36 | 66.30 | 104.70 | 100.16 | 69.85 |
| Marine eutrophication | kg N eq. | 21.78 | 22.97 | 22.59 | 21.08 | 20.95 | 21.95 |
| Mineral resource depletion | kg Fe eq. | 112.70 | 58.55 | 58.55 | 61.22 | 60.90 | 58.80 |
| Natural land transformation | m^2^ | -5.62 | -4.36 | -4.36 | -7.26 | -6.91 | -4.63 |
| Ozone depletion | g CFC-11 eq. | -2.80 | -2.17 | -2.16 | -3.61 | -3.44 | -2.30 |
| Particulate matter formation | kg PM_10_ eq. | 4.53 | 3.22 | 3.22 | 3.97 | 3.88 | 3.29 |
| Photochemical oxidant formation | kg NMVOC | 8.22 | 5.97 | 5.97 | 7.90 | 7.67 | 6.15 |
| Terrestrial acidification | kg SO_2_ eq. | -2.94 | -1.65 | -1.64 | -7.87 | -7.13 | -2.22 |
| Terrestrial ecotoxicity | kg 1,4-DB eq. | 1.62 | 1.59 | 1.59 | 1.60 | 1.60 | 1.59 |
| Urban land occupation | m^2^*a | 26.46 | 11.64 | 11.63 | 12.94 | 12.78 | 11.76 |
| Water depletion | m^3^ | 5859.47 | 2597.69 | 2595.75 | 3690.61 | 3561.12 | 2697.12 |
| **Results LCIA** | **Reference unit** | **Locations [results per MJ_th_]** | | | | | |
|  |  | **Adana** | **Aberystwyth** | **Moscow** | **Potash** | **Stuttgart** | **Wageningen** |
| Agricultural land occupation | m^2^*a | 5.93E-02 | 7.60E-02 | 7.61E-02 | 4.62E-02 | 4.85E-02 | 7.18E-02 |
| Climate Change | kg CO_2_ eq. | -7.60E-02 | -7.64E-02 | -7.64E-02 | -7.91E-02 | -7.89E-02 | -7.68E-02 |
| Fossil fuel depletion | kg oil eq. | -2.88E-02 | -2.91E-02 | -2.91E-02 | -2.96E-02 | -2.96E-02 | -2.92E-02 |
| Freshwater ecotoxicity | kg 1.4-DB eq. | 5.57E-04 | 5.21E-04 | 5.22E-04 | 4.98E-04 | 5.00E-04 | 5.18E-04 |
| Freshwater eutrophication | kg P eq. | 7.21E-06 | 7.71E-06 | 7.71E-06 | 4.86E-06 | 5.08E-06 | 7.30E-06 |
| Human toxicity | kg 1.4-DB eq. | 4.38E-02 | 4.36E-02 | 4.36E-02 | 4.22E-02 | 4.23E-02 | 4.34E-02 |
| Ionising radiation | kg U235 eq. | -5.56E-03 | -5.77E-03 | -5.77E-03 | -5.85E-03 | -5.85E-03 | -5.78E-03 |
| Marine ecotoxicity | kg 1.4-DB eq. | 5.31E-04 | 4.99E-04 | 4.99E-04 | 4.77E-04 | 4.79E-04 | 4.96E-04 |
| Marine eutrophication | kg N eq. | 1.27E-04 | 1.73E-04 | 1.70E-04 | 9.61E-05 | 1.00E-04 | 1.56E-04 |
| Mineral resource depletion | kg Fe eq. | 6.55E-04 | 4.40E-04 | 4.40E-04 | 2.79E-04 | 2.91E-04 | 4.17E-04 |
| Natural land transformation | m^2^ | -3.27E-05 | -3.28E-05 | -3.28E-05 | -3.31E-05 | -3.31E-05 | -3.28E-05 |
| Ozone depletion | kg CFC-11 eq. | -1.63E-08 | -1.63E-08 | -1.63E-08 | -1.65E-08 | -1.64E-08 | -1.63E-08 |
| Particulate matter formation | kg PM_10_ eq. | 2.63E-05 | 2.42E-05 | 2.42E-05 | 1.81E-05 | 1.85E-05 | 2.33E-05 |
| Photochemical oxidant formation | kg NMVOC | 4.78E-05 | 4.49E-05 | 4.49E-05 | 3.60E-05 | 3.67E-05 | 4.36E-05 |
| Terrestrial acidification | kg SO_2_ eq. | -1.71E-05 | -1.24E-05 | -1.23E-05 | -3.59E-05 | -3.41E-05 | -1.57E-05 |
| Terrestrial ecotoxicity | kg 1.4-DB eq. | 9.40E-06 | 1.19E-05 | 1.19E-05 | 7.31E-06 | 7.66E-06 | 1.13E-05 |
| Urban land occupation | m^2^*a | 1.54E-04 | 8.74E-05 | 8.75E-05 | 5.90E-05 | 6.11E-05 | 8.34E-05 |
| Water depletion | m^3^ | 3.40E-02 | 1.95E-02 | 1.95E-02 | 1.68E-02 | 1.70E-02 | 1.91E-02 |
