# Supplementary material for: Novel Miscanthus Germplasm-Based Value Chains: A Life Cycle Assessment
Source: Front Plant Sci. 2017 Jun 8;8:990. doi: 10.3389/fpls.2017.00990 (PMC5462955; doi:10.3389/fpls.2017.00990)
Supplement: Supplementary file 3 [file Table3.DOCX]

Table S3: Environmental benefits and impacts per ha and MJ_th_ for utilization pathway 2 [Small-scale combustion – pellets]

| **Results LCIA** | **Reference unit** | **Locations [results per ha]** | | | | | |
| --- | --- | --- | --- | --- | --- | --- | --- |
|  |  | **Adana** | **Aberystwyth** | **Moscow** | **Potash** | **Stuttgart** | **Wageningen** |
| Agricultural land occupation | m^2^*a | 10281.56 | 10171.69 | 10171.59 | 10228.89 | 10222.11 | 10176.89 |
| Climate Change | kg CO_2_ eq. | -12092.05 | -9399.19 | -9388.58 | -16100.53 | -15307.76 | -10011.70 |
| Fossil fuel depletion | kg oil eq. | -4650.68 | -3645.17 | -3640.78 | -6116.29 | -5823.50 | -3869.97 |
| Freshwater ecotoxicity | kg 1,4-DB eq. | 108.84 | 79.51 | 79.43 | 126.01 | 120.50 | 83.74 |
| Freshwater eutrophication | kg P eq. | 1.79 | 1.45 | 1.45 | 1.77 | 1.73 | 1.48 |
| Human toxicity | kg 1,4-DB eq. | 7985.84 | 6152.65 | 6146.10 | 9840.66 | 9403.69 | 6488.15 |
| Ionising radiation | kg U235 eq. | -628.65 | -514.03 | -513.41 | -865.20 | -823.59 | -545.98 |
| Marine ecotoxicity | kg 1,4-DB eq. | 104.82 | 76.81 | 76.73 | 121.92 | 116.58 | 80.91 |
| Marine eutrophication | kg N eq. | 21.99 | 23.13 | 22.75 | 21.35 | 21.20 | 22.12 |
| Mineral resource depletion | kg Fe eq. | 121.79 | 65.58 | 65.57 | 72.81 | 71.95 | 66.24 |
| Natural land transformation | m^2^ | -5.40 | -4.19 | -4.19 | -6.98 | -6.65 | -4.45 |
| Ozone depletion | g CFC-11 eq. | -2.66 | -2.06 | -2.05 | -3.43 | -3.27 | -2.18 |
| Particulate matter formation | kg PM_10_ eq. | 5.80 | 4.20 | 4.19 | 5.58 | 5.42 | 4.32 |
| Photochemical oxidant formation | kg NMVOC | 10.50 | 7.74 | 7.73 | 10.81 | 10.44 | 8.02 |
| Terrestrial acidification | kg SO_2_ eq. | 0.53 | 1.03 | 1.04 | -3.45 | -2.92 | 0.62 |
| Terrestrial ecotoxicity | kg 1,4-DB eq. | 1.86 | 1.77 | 1.77 | 1.91 | 1.90 | 1.79 |
| Urban land occupation | m^2^*a | 51.63 | 31.10 | 31.08 | 45.03 | 43.38 | 32.37 |
| Water depletion | m^3^ | 11227.08 | 6748.31 | 6741.58 | 10532.93 | 10084.51 | 7092.60 |
| **Results LCIA** | **Reference unit** | **Locations [results per MJ_th_]** | | | | | |
|  |  | **Adana** | **Aberystwyth** | **Moscow** | **Potash** | **Stuttgart** | **Wageningen** |
| Agricultural land occupation | m^2^*a | 5.97E-02 | 7.64E-02 | 7.65E-02 | 4.66E-02 | 4.89E-02 | 7.22E-02 |
| Climate Change | kg CO_2_ eq. | -7.03E-02 | -7.06E-02 | -7.06E-02 | -7.34E-02 | -7.32E-02 | -7.10E-02 |
| Fossil fuel depletion | kg oil eq. | -2.70E-02 | -2.74E-02 | -2.74E-02 | -2.79E-02 | -2.78E-02 | -2.75E-02 |
| Freshwater ecotoxicity | kg 1.4-DB eq. | 6.32E-04 | 5.97E-04 | 5.97E-04 | 5.74E-04 | 5.76E-04 | 5.94E-04 |
| Freshwater eutrophication | kg P eq. | 1.04E-05 | 1.09E-05 | 1.09E-05 | 8.07E-06 | 8.28E-06 | 1.05E-05 |
| Human toxicity | kg 1.4-DB eq. | 4.64E-02 | 4.62E-02 | 4.62E-02 | 4.48E-02 | 4.50E-02 | 4.60E-02 |
| Ionising radiation | kg U235 eq. | -3.65E-03 | -3.86E-03 | -3.86E-03 | -3.94E-03 | -3.94E-03 | -3.87E-03 |
| Marine ecotoxicity | kg 1.4-DB eq. | 6.09E-04 | 5.77E-04 | 5.77E-04 | 5.56E-04 | 5.57E-04 | 5.74E-04 |
| Marine eutrophication | kg N eq. | 1.28E-04 | 1.74E-04 | 1.71E-04 | 9.73E-05 | 1.01E-04 | 1.57E-04 |
| Mineral resource depletion | kg Fe eq. | 7.08E-04 | 4.93E-04 | 4.93E-04 | 3.32E-04 | 3.44E-04 | 4.70E-04 |
| Natural land transformation | m^2^ | -3.14E-05 | -3.15E-05 | -3.15E-05 | -3.18E-05 | -3.18E-05 | -3.15E-05 |
| Ozone depletion | kg CFC-11 eq. | -1.54E-08 | -1.55E-08 | -1.55E-08 | -1.56E-08 | -1.56E-08 | -1.55E-08 |
| Particulate matter formation | kg PM_10_ eq. | 3.37E-05 | 3.15E-05 | 3.15E-05 | 2.54E-05 | 2.59E-05 | 3.07E-05 |
| Photochemical oxidant formation | kg NMVOC | 6.10E-05 | 5.81E-05 | 5.82E-05 | 4.93E-05 | 4.99E-05 | 5.69E-05 |
| Terrestrial acidification | kg SO_2_ eq. | 3.07E-06 | 7.73E-06 | 7.80E-06 | -1.57E-05 | -1.40E-05 | 4.41E-06 |
| Terrestrial ecotoxicity | kg 1.4-DB eq. | 1.08E-05 | 1.33E-05 | 1.33E-05 | 8.72E-06 | 9.07E-06 | 1.27E-05 |
| Urban land occupation | m^2^*a | 3.00E-04 | 2.34E-04 | 2.34E-04 | 2.05E-04 | 2.07E-04 | 2.30E-04 |
| Water depletion | m^3^ | 6.52E-02 | 5.07E-02 | 5.07E-02 | 4.80E-02 | 4.82E-02 | 5.03E-02 |
