# Supplementary material for: Novel Miscanthus Germplasm-Based Value Chains: A Life Cycle Assessment
Source: Front Plant Sci. 2017 Jun 8;8:990. doi: 10.3389/fpls.2017.00990 (PMC5462955; doi:10.3389/fpls.2017.00990)
Supplement: Supplementary file 4 [file Table4.DOCX]

Table S4: Environmental benefits and impacts per ha and MJ_th_ for utilization pathway 3 [Large-scale combustion – biomass baled for transport and storage]

| **Results LCIA** | **Reference unit** | **Locations [results per ha]** | | | | | |
| --- | --- | --- | --- | --- | --- | --- | --- |
|  |  | **Adana** | **Aberystwyth** | **Moscow** | **Potash** | **Stuttgart** | **Wageningen** |
| Agricultural land occupation | m^2^*a | 9611.66 | 9655.36 | 9655.85 | 9376.57 | 9409.60 | 9630.00 |
| Climate Change | kg CO_2_ eq. | -5282.12 | -4131.94 | -4127.41 | -7417.56 | -7029.51 | -4433.73 |
| Fossil fuel depletion | kg oil eq. | -1899.54 | -1517.34 | -1515.40 | -2608.56 | -2479.26 | -1616.60 |
| Freshwater ecotoxicity | kg 1,4-DB eq. | 13.95 | 6.13 | 6.13 | 5.03 | 5.16 | 6.03 |
| Freshwater eutrophication | kg P eq. | -2.86 | -2.14 | -2.14 | -4.16 | -3.92 | -2.33 |
| Human toxicity | kg 1,4-DB eq. | 5326.33 | 4095.91 | 4091.73 | 6450.07 | 6171.14 | 4310.07 |
| Ionising radiation | kg U235 eq. | -2094.93 | -1648.01 | -1646.08 | -2734.58 | -2605.84 | -1746.86 |
| Marine ecotoxicity | kg 1,4-DB eq. | 15.23 | 7.52 | 7.52 | 7.70 | 7.68 | 7.54 |
| Marine eutrophication | kg N eq. | 21.04 | 22.40 | 22.02 | 20.15 | 20.05 | 21.35 |
| Mineral resource depletion | kg Fe eq. | 142.74 | 81.81 | 81.77 | 99.55 | 97.44 | 83.42 |
| Natural land transformation | m^2^ | -0.80 | -0.64 | -0.64 | -1.12 | -1.06 | -0.68 |
| Ozone depletion | g CFC-11 eq. | 0.49 | 0.38 | 0.38 | 0.58 | 0.56 | 0.40 |
| Particulate matter formation | kg PM_10_ eq. | 2.70 | 1.80 | 1.80 | 1.63 | 1.65 | 1.79 |
| Photochemical oxidant formation | kg NMVOC | 7.75 | 5.60 | 5.60 | 7.29 | 7.09 | 5.76 |
| Terrestrial acidification | kg SO_2_ eq. | 7.45 | 6.38 | 6.39 | 5.38 | 5.50 | 6.29 |
| Terrestrial ecotoxicity | kg 1,4-DB eq. | 2.04 | 1.91 | 1.91 | 2.14 | 2.11 | 1.93 |
| Urban land occupation | m^2^*a | 45.89 | 26.67 | 26.65 | 37.72 | 36.41 | 27.68 |
| Water depletion | m^3^ | -35852.08 | -29661.94 | -29626.69 | -49489.63 | -47140.37 | -31465.66 |
| **Results LCIA** | **Reference unit** | **Locations [results per MJ_th_]** | | | | | |
|  |  | **Adana** | **Aberystwyth** | **Moscow** | **Potash** | **Stuttgart** | **Wageningen** |
| Agricultural land occupation | m^2^*a | 9.31E-02 | 1.21E-01 | 1.21E-01 | 7.12E-02 | 7.50E-02 | 1.14E-01 |
| Climate Change | kg CO_2_ eq. | -5.12E-02 | -5.17E-02 | -5.17E-02 | -5.63E-02 | -5.60E-02 | -5.24E-02 |
| Fossil fuel depletion | kg oil eq. | -1.84E-02 | -1.90E-02 | -1.90E-02 | -1.98E-02 | -1.98E-02 | -1.91E-02 |
| Freshwater ecotoxicity | kg 1.4-DB eq. | 1.35E-04 | 7.67E-05 | 7.68E-05 | 3.82E-05 | 4.11E-05 | 7.13E-05 |
| Freshwater eutrophication | kg P eq. | -2.77E-05 | -2.69E-05 | -2.68E-05 | -3.16E-05 | -3.12E-05 | -2.75E-05 |
| Human toxicity | kg 1.4-DB eq. | 5.16E-02 | 5.13E-02 | 5.13E-02 | 4.90E-02 | 4.92E-02 | 5.10E-02 |
| Ionising radiation | kg U235 eq. | -2.03E-02 | -2.06E-02 | -2.06E-02 | -2.08E-02 | -2.08E-02 | -2.07E-02 |
| Marine ecotoxicity | kg 1.4-DB eq. | 1.47E-04 | 9.42E-05 | 9.43E-05 | 5.85E-05 | 6.12E-05 | 8.92E-05 |
| Marine eutrophication | kg N eq. | 2.04E-04 | 2.80E-04 | 2.76E-04 | 1.53E-04 | 1.60E-04 | 2.52E-04 |
| Mineral resource depletion | kg Fe eq. | 1.38E-03 | 1.02E-03 | 1.03E-03 | 7.56E-04 | 7.76E-04 | 9.86E-04 |
| Natural land transformation | m^2^ | -7.79E-06 | -7.99E-06 | -7.99E-06 | -8.48E-06 | -8.45E-06 | -8.06E-06 |
| Ozone depletion | kg CFC-11 eq. | 4.77E-09 | 4.73E-09 | 4.73E-09 | 4.43E-09 | 4.45E-09 | 4.69E-09 |
| Particulate matter formation | kg PM_10_ eq. | 2.62E-05 | 2.25E-05 | 2.26E-05 | 1.24E-05 | 1.32E-05 | 2.11E-05 |
| Photochemical oxidant formation | kg NMVOC | 7.50E-05 | 7.02E-05 | 7.02E-05 | 5.54E-05 | 5.65E-05 | 6.81E-05 |
| Terrestrial acidification | kg SO_2_ eq. | 7.21E-05 | 7.99E-05 | 8.01E-05 | 4.08E-05 | 4.38E-05 | 7.44E-05 |
| Terrestrial ecotoxicity | kg 1.4-DB eq. | 1.97E-05 | 2.39E-05 | 2.39E-05 | 1.62E-05 | 1.68E-05 | 2.28E-05 |
| Urban land occupation | m^2^*a | 4.44E-04 | 3.34E-04 | 3.34E-04 | 2.87E-04 | 2.90E-04 | 3.27E-04 |
| Water depletion | m^3^ | -3.47E-01 | -3.71E-01 | -3.71E-01 | -3.76E-01 | -3.76E-01 | -3.72E-01 |
