# Supplementary material for: Novel Miscanthus Germplasm-Based Value Chains: A Life Cycle Assessment
Source: Front Plant Sci. 2017 Jun 8;8:990. doi: 10.3389/fpls.2017.00990 (PMC5462955; doi:10.3389/fpls.2017.00990)
Supplement: Supplementary file 5 [file Table5.DOCX]

Table S5: Environmental benefits and impacts per ha and MJ_th_ for utilization pathway 4 [Large-scale combustion – pellets]

| **Results LCIA** | **Reference unit** | **Locations [results per ha]** | | | | | |
| --- | --- | --- | --- | --- | --- | --- | --- |
|  |  | **Adana** | **Aberystwyth** | **Moscow** | **Potash** | **Stuttgart** | **Wageningen** |
| Agricultural land occupation | m^2^*a | 9690.55 | 9716.38 | 9716.80 | 9477.16 | 9505.50 | 9694.61 |
| Climate Change | kg CO_2_ eq. | -4860.12 | -3805.57 | -3801.41 | -6879.53 | -6516.55 | -4088.11 |
| Fossil fuel depletion | kg oil eq. | -1812.85 | -1450.29 | -1448.43 | -2498.04 | -2373.89 | -1545.61 |
| Freshwater ecotoxicity | kg 1,4-DB eq. | 25.27 | 14.88 | 14.87 | 19.46 | 18.92 | 15.29 |
| Freshwater eutrophication | kg P eq. | -2.32 | -1.73 | -1.73 | -3.48 | -3.27 | -1.89 |
| Human toxicity | kg 1,4-DB eq. | 5632.82 | 4332.96 | 4328.50 | 6840.83 | 6543.69 | 4561.10 |
| Ionising radiation | kg U235 eq. | -1819.03 | -1434.63 | -1432.94 | -2382.81 | -2270.47 | -1520.88 |
| Marine ecotoxicity | kg 1,4-DB eq. | 25.24 | 15.27 | 15.26 | 20.47 | 19.85 | 15.74 |
| Marine eutrophication | kg N eq. | 21.18 | 22.51 | 22.14 | 20.33 | 20.23 | 21.47 |
| Mineral resource depletion | kg Fe eq. | 143.13 | 82.11 | 82.08 | 100.04 | 97.92 | 83.74 |
| Natural land transformation | m^2^ | -0.83 | -0.66 | -0.66 | -1.15 | -1.09 | -0.70 |
| Ozone depletion | g CFC-11 eq. | 0.52 | 0.40 | 0.40 | 0.61 | 0.59 | 0.42 |
| Particulate matter formation | kg PM_10_ eq. | 3.21 | 2.19 | 2.19 | 2.28 | 2.27 | 2.20 |
| Photochemical oxidant formation | kg NMVOC | 8.15 | 5.92 | 5.91 | 7.81 | 7.58 | 6.09 |
| Terrestrial acidification | kg SO_2_ eq. | 9.49 | 7.96 | 7.96 | 7.97 | 7.97 | 7.96 |
| Terrestrial ecotoxicity | kg 1,4-DB eq. | 1.95 | 1.85 | 1.85 | 2.03 | 2.01 | 1.86 |
| Urban land occupation | m^2^*a | 37.18 | 19.94 | 19.93 | 26.62 | 25.83 | 20.55 |
| Water depletion | m^3^ | -30692.31 | -25671.35 | -25640.70 | -42911.12 | -40868.48 | -27239.64 |
| **Results LCIA** | **Reference unit** | **Locations [results per MJ_th_]** | | | | | |
|  |  | **Adana** | **Aberystwyth** | **Moscow** | **Potash** | **Stuttgart** | **Wageningen** |
| Agricultural land occupation | m^2^*a | 9.38E-02 | 1.22E-01 | 1.22E-01 | 7.20E-02 | 7.57E-02 | 1.15E-01 |
| Climate Change | kg CO_2_ eq. | -4.71E-02 | -4.77E-02 | -4.77E-02 | -5.23E-02 | -5.19E-02 | -4.83E-02 |
| Fossil fuel depletion | kg oil eq. | -1.76E-02 | -1.82E-02 | -1.82E-02 | -1.90E-02 | -1.89E-02 | -1.83E-02 |
| Freshwater ecotoxicity | kg 1.4-DB eq. | 2.45E-04 | 1.86E-04 | 1.86E-04 | 1.48E-04 | 1.51E-04 | 1.81E-04 |
| Freshwater eutrophication | kg P eq. | -2.25E-05 | -2.17E-05 | -2.16E-05 | -2.64E-05 | -2.60E-05 | -2.23E-05 |
| Human toxicity | kg 1.4-DB eq. | 5.45E-02 | 5.43E-02 | 5.43E-02 | 5.20E-02 | 5.21E-02 | 5.39E-02 |
| Ionising radiation | kg U235 eq. | -1.76E-02 | -1.80E-02 | -1.80E-02 | -1.81E-02 | -1.81E-02 | -1.80E-02 |
| Marine ecotoxicity | kg 1.4-DB eq. | 2.44E-04 | 1.91E-04 | 1.91E-04 | 1.55E-04 | 1.58E-04 | 1.86E-04 |
| Marine eutrophication | kg N eq. | 2.05E-04 | 2.82E-04 | 2.77E-04 | 1.54E-04 | 1.61E-04 | 2.54E-04 |
| Mineral resource depletion | kg Fe eq. | 1.39E-03 | 1.03E-03 | 1.03E-03 | 7.60E-04 | 7.80E-04 | 9.90E-04 |
| Natural land transformation | m^2^ | -8.04E-06 | -8.24E-06 | -8.24E-06 | -8.73E-06 | -8.70E-06 | -8.31E-06 |
| Ozone depletion | kg CFC-11 eq. | 5.00E-09 | 4.96E-09 | 4.96E-09 | 4.66E-09 | 4.68E-09 | 4.92E-09 |
| Particulate matter formation | kg PM_10_ eq. | 3.11E-05 | 2.75E-05 | 2.75E-05 | 1.73E-05 | 1.81E-05 | 2.60E-05 |
| Photochemical oxidant formation | kg NMVOC | 7.89E-05 | 7.41E-05 | 7.41E-05 | 5.93E-05 | 6.04E-05 | 7.20E-05 |
| Terrestrial acidification | kg SO_2_ eq. | 9.19E-05 | 9.97E-05 | 9.98E-05 | 6.06E-05 | 6.35E-05 | 9.41E-05 |
| Terrestrial ecotoxicity | kg 1.4-DB eq. | 1.89E-05 | 2.31E-05 | 2.31E-05 | 1.54E-05 | 1.60E-05 | 2.20E-05 |
| Urban land occupation | m^2^*a | 3.60E-04 | 2.50E-04 | 2.50E-04 | 2.02E-04 | 2.06E-04 | 2.43E-04 |
| Water depletion | m^3^ | -2.97E-01 | -3.21E-01 | -3.21E-01 | -3.26E-01 | -3.26E-01 | -3.22E-01 |
