# Supplementary material for: Novel Miscanthus Germplasm-Based Value Chains: A Life Cycle Assessment
Source: Front Plant Sci. 2017 Jun 8;8:990. doi: 10.3389/fpls.2017.00990 (PMC5462955; doi:10.3389/fpls.2017.00990)
Supplement: Supplementary file 6 [file Table6.DOCX]

Table S6: Environmental benefits and impacts per ha and MJ_el_ for utilization pathway 5 [Medium-scale biogas plant – biomass ensiled]

| **Results LCIA** | **Reference unit** | **Locations [results per ha]** | | |
| --- | --- | --- | --- | --- |
|  |  | **Adana** | **Moscow** | **Stuttgart** |
| Agricultural land occupation | m^2^*a | 9449.35 | 9437.43 | 9043.08 |
| Climate Change | kg CO_2_ eq. | -5252.46 | -4797.38 | -9140.67 |
| Fossil fuel depletion | kg oil eq. | -1611.34 | -1513.71 | -2610.91 |
| Freshwater ecotoxicity | kg 1,4-DB eq. | -72.06 | -70.53 | -120.71 |
| Freshwater eutrophication | kg P eq. | -4.47 | -4.05 | -6.99 |
| Human toxicity | kg 1,4-DB eq. | -2896.50 | -2617.45 | -4721.25 |
| Ionising radiation | kg U235 eq. | -3017.13 | -2739.78 | -4288.73 |
| Marine ecotoxicity | kg 1,4-DB eq. | -70.06 | -68.13 | -116.84 |
| Marine eutrophication | kg N eq. | 24.47 | 22.18 | 23.42 |
| Mineral resource depletion | kg Fe eq. | 178.88 | 123.03 | 91.66 |
| Natural land transformation | m^2^ | -0.56 | -0.53 | -1.00 |
| Ozone depletion | g CFC-11 eq. | -0.67 | -0.61 | -1.04 |
| Particulate matter formation | kg PM_10_ eq. | -1.82 | -2.12 | -6.46 |
| Photochemical oxidant formation | kg NMVOC | -0.58 | -1.41 | -6.96 |
| Terrestrial acidification | kg SO_2_ eq. | -5.24 | -4.19 | -18.61 |
| Terrestrial ecotoxicity | kg 1,4-DB eq. | 1.50 | 1.57 | 1.27 |
| Urban land occupation | m^2^*a | 6.90 | -4.70 | -23.81 |
| Water depletion | m^3^ | -51006.37 | -48082.75 | -76049.34 |
| **Results LCIA** | **Reference unit** | **Locations [results per MJ_el_]** | | |
|  |  | **Adana** | **Moscow** | **Stuttgart** |
| Agricultural land occupation | m^2^*a | 1.91E-01 | 2.13E-01 | 1.32E-01 |
| Climate Change | kg CO_2_ eq. | -1.06E-01 | -1.08E-01 | -1.33E-01 |
| Fossil fuel depletion | kg oil eq. | -3.26E-02 | -3.41E-02 | -3.80E-02 |
| Freshwater ecotoxicity | kg 1.4-DB eq. | -1.46E-03 | -1.59E-03 | -1.76E-03 |
| Freshwater eutrophication | kg P eq. | -9.02E-05 | -9.13E-05 | -1.02E-04 |
| Human toxicity | kg 1.4-DB eq. | -5.85E-02 | -5.90E-02 | -6.87E-02 |
| Ionising radiation | kg U235 eq. | -6.10E-02 | -6.17E-02 | -6.24E-02 |
| Marine ecotoxicity | kg 1.4-DB eq. | -1.42E-03 | -1.53E-03 | -1.70E-03 |
| Marine eutrophication | kg N eq. | 4.94E-04 | 5.00E-04 | 3.41E-04 |
| Mineral resource depletion | kg Fe eq. | 3.61E-03 | 2.77E-03 | 1.33E-03 |
| Natural land transformation | m^2^ | -1.12E-05 | -1.18E-05 | -1.46E-05 |
| Ozone depletion | kg CFC-11 eq. | -1.35E-08 | -1.37E-08 | -1.51E-08 |
| Particulate matter formation | kg PM_10_ eq. | -3.68E-05 | -4.77E-05 | -9.39E-05 |
| Photochemical oxidant formation | kg NMVOC | -1.16E-05 | -3.17E-05 | -1.01E-04 |
| Terrestrial acidification | kg SO_2_ eq. | -1.06E-04 | -9.43E-05 | -2.71E-04 |
| Terrestrial ecotoxicity | kg 1.4-DB eq. | 3.03E-05 | 3.53E-05 | 1.85E-05 |
| Urban land occupation | m^2^*a | 1.39E-04 | -1.06E-04 | -3.46E-04 |
| Water depletion | m^3^ | -1.03E+00 | -1.08E+00 | -1.11E+00 |
