# Supplementary material for: Novel Miscanthus Germplasm-Based Value Chains: A Life Cycle Assessment
Source: Front Plant Sci. 2017 Jun 8;8:990. doi: 10.3389/fpls.2017.00990 (PMC5462955; doi:10.3389/fpls.2017.00990)
Supplement: Supplementary file 7 [file Table7.DOCX]

Table S7: Environmental benefits and impacts per ha and per m³ insulation material for utilization pathway 6 [Large-scale production of insulation material – biomass baled for transport and storage]

| **Results LCIA** | **Reference unit** | **Locations [results per ha]** | | | | | |
| --- | --- | --- | --- | --- | --- | --- | --- |
|  |  | **Adana** | **Aberystwyth** | **Moscow** | **Potash** | **Stuttgart** | **Wageningen** |
| Agricultural land occupation | m^2^*a | 8848.20 | 9064.89 | 9066.07 | 8403.19 | 8481.59 | 9004.70 |
| Climate Change | kg CO_2_ eq. | -18624.35 | -14450.90 | -14434.47 | -24428.41 | -23247.47 | -15361.45 |
| Fossil fuel depletion | kg oil eq. | -5988.37 | -4679.66 | -4674.08 | -7821.67 | -7449.39 | -4965.49 |
| Freshwater ecotoxicity | kg 1,4-DB eq. | -22.81 | -22.31 | -22.27 | -41.84 | -39.53 | -24.09 |
| Freshwater eutrophication | kg P eq. | -10.39 | -7.97 | -7.96 | -13.76 | -13.07 | -8.49 |
| Human toxicity | kg 1,4-DB eq. | 226.27 | 151.50 | 151.86 | -52.32 | -28.17 | 132.96 |
| Ionising radiation | kg U235 eq. | -3985.71 | -3110.34 | -3106.73 | -5145.24 | -4904.14 | -3295.46 |
| Marine ecotoxicity | kg 1,4-DB eq. | -2.89 | -6.48 | -6.47 | -15.39 | -14.34 | -7.29 |
| Marine eutrophication | kg N eq. | 15.81 | 18.36 | 17.99 | 13.48 | 13.70 | 17.07 |
| Mineral resource depletion | kg Fe eq. | -2068.19 | -1628.15 | -1626.21 | -2719.32 | -2590.03 | -1727.41 |
| Natural land transformation | m^2^ | -2.35 | -1.83 | -1.83 | -3.09 | -2.94 | -1.95 |
| Ozone depletion | g CFC-11 eq. | -0.69 | -0.53 | -0.53 | -0.92 | -0.88 | -0.57 |
| Particulate matter formation | kg PM_10_ eq. | -48.61 | -37.88 | -37.84 | -63.79 | -60.72 | -40.24 |
| Photochemical oxidant formation | kg NMVOC | -56.55 | -44.12 | -44.06 | -74.68 | -71.06 | -46.90 |
| Terrestrial acidification | kg SO_2_ eq. | -95.29 | -73.07 | -72.98 | -125.61 | -119.38 | -77.85 |
| Terrestrial ecotoxicity | kg 1,4-DB eq. | -0.21 | 0.17 | 0.17 | -0.73 | -0.62 | 0.09 |
| Urban land occupation | m^2^*a | -52.89 | -49.73 | -49.66 | -88.22 | -83.66 | -53.23 |
| Water depletion | m^3^ | -123645.89 | -97562.19 | -97448.67 | -161423.49 | -153856.94 | -103371.63 |
| **Results LCIA** | **Reference unit** | **Locations [results per m³ insulation material]** | | | | | |
|  |  | **Adana** | **Aber** | **Moscow** | **Potash** | **Stuttgart** | **Wageningen** |
| Agricultural land occupation | m^2^*a | 1.36E+02 | 1.81E+02 | 1.81E+02 | 1.02E+02 | 1.08E+02 | 1.70E+02 |
| Climate Change | kg CO_2_ eq. | -2.87E+02 | -2.88E+02 | -2.88E+02 | -2.95E+02 | -2.95E+02 | -2.89E+02 |
| Fossil fuel depletion | kg oil eq. | -9.23E+01 | -9.33E+01 | -9.33E+01 | -9.46E+01 | -9.45E+01 | -9.35E+01 |
| Freshwater ecotoxicity | kg 1.4-DB eq. | -3.52E-01 | -4.45E-01 | -4.45E-01 | -5.06E-01 | -5.01E-01 | -4.53E-01 |
| Freshwater eutrophication | kg P eq. | -1.60E-01 | -1.59E-01 | -1.59E-01 | -1.66E-01 | -1.66E-01 | -1.60E-01 |
| Human toxicity | kg 1.4-DB eq. | 3.49E+00 | 3.02E+00 | 3.03E+00 | -6.33E-01 | -3.57E-01 | 2.50E+00 |
| Ionising radiation | kg U235 eq. | -6.15E+01 | -6.20E+01 | -6.20E+01 | -6.22E+01 | -6.22E+01 | -6.20E+01 |
| Marine ecotoxicity | kg 1.4-DB eq. | -4.45E-02 | -1.29E-01 | -1.29E-01 | -1.86E-01 | -1.82E-01 | -1.37E-01 |
| Marine eutrophication | kg N eq. | 2.44E-01 | 3.66E-01 | 3.59E-01 | 1.63E-01 | 1.74E-01 | 3.21E-01 |
| Mineral resource depletion | kg Fe eq. | -3.19E+01 | -3.25E+01 | -3.25E+01 | -3.29E+01 | -3.29E+01 | -3.25E+01 |
| Natural land transformation | m^2^ | -3.62E-02 | -3.65E-02 | -3.65E-02 | -3.73E-02 | -3.73E-02 | -3.66E-02 |
| Ozone depletion | kg CFC-11 eq. | -1.06E-05 | -1.07E-05 | -1.07E-05 | -1.11E-05 | -1.11E-05 | -1.07E-05 |
| Particulate matter formation | kg PM_10_ eq. | -7.50E-01 | -7.55E-01 | -7.55E-01 | -7.71E-01 | -7.70E-01 | -7.58E-01 |
| Photochemical oxidant formation | kg NMVOC | -8.72E-01 | -8.80E-01 | -8.80E-01 | -9.03E-01 | -9.01E-01 | -8.83E-01 |
| Terrestrial acidification | kg SO_2_ eq. | -1.47E+00 | -1.46E+00 | -1.46E+00 | -1.52E+00 | -1.51E+00 | -1.47E+00 |
| Terrestrial ecotoxicity | kg 1.4-DB eq. | -3.30E-03 | 3.37E-03 | 3.41E-03 | -8.85E-03 | -7.92E-03 | 1.64E-03 |
| Urban land occupation | m^2^*a | -8.16E-01 | -9.91E-01 | -9.91E-01 | -1.07E+00 | -1.06E+00 | -1.00E+00 |
| Water depletion | m^3^ | -1.91E+03 | -1.95E+03 | -1.95E+03 | -1.95E+03 | -1.95E+03 | -1.95E+03 |
