# Supplementary material for: Novel Miscanthus Germplasm-Based Value Chains: A Life Cycle Assessment
Source: Front Plant Sci. 2017 Jun 8;8:990. doi: 10.3389/fpls.2017.00990 (PMC5462955; doi:10.3389/fpls.2017.00990)
Supplement: Supplementary file 8 [file Table8.DOCX]

Table S8: Influence of the fossil reference on the final results of utilization pathway 1 (small-scale combustion - chips)

| **Results Per ha (Stuttgart)** | **Reference unit** | **Fossil reference** | |
| --- | --- | --- | --- |
|  |  | **Light fuel oil** | **Natural gas** |
| Agricultural land occupation | m^2^*a | 10140.09 | 10192.66 |
| Climate Change | kg CO_2_ eq. | -16515.00 | -3795.21 |
| Fossil fuel depletion | kg oil eq. | -6192.41 | -2068.81 |
| Freshwater ecotoxicity | kg 1.4-DB eq. | 104.63 | 125.35 |
| Freshwater eutrophication | kg P eq. | 1.06 | 1.35 |
| Human toxicity | kg 1.4-DB eq. | 8859.15 | 9471.74 |
| Ionising radiation | kg U235 eq. | -1223.17 | 19.45 |
| Marine ecotoxicity | kg 1.4-DB eq. | 100.16 | 117.36 |
| Marine eutrophication | kg N eq. | 20.95 | 21.54 |
| Mineral resource depletion | kg Fe eq. | 60.90 | 201.35 |
| Natural land transformation | m^2^ | -6.91 | -1.22 |
| Ozone depletion | g CFC-11 eq. | -3.44 | -0.92 |
| Particulate matter formation | kg PM_10_ eq. | 3.88 | 12.40 |
| Photochemical oxidant formation | kg NMVOC | 7.67 | 24.66 |
| Terrestrial acidification | kg SO_2_ eq. | -7.13 | 25.51 |
| Terrestrial ecotoxicity | kg 1.4-DB eq. | 1.60 | 1.92 |
| Urban land occupation | m^2^*a | 12.78 | 38.13 |
| Water depletion | m^3^ | 3561.12 | 5134.68 |
