# Supplementary material for: Novel Miscanthus Germplasm-Based Value Chains: A Life Cycle Assessment
Source: Front Plant Sci. 2017 Jun 8;8:990. doi: 10.3389/fpls.2017.00990 (PMC5462955; doi:10.3389/fpls.2017.00990)
Supplement: Supplementary file 9 [file Table9.DOCX]

Table S9: Comparison of credits given and the substitution effect of the fossil reference in case of the utilization pathways 3 and 4

| **Results per ha** | **Reference unit** | **Emission Credits** | **Fossil reference** |
| --- | --- | --- | --- |
|  |  | **Per 1/3 MJ_el_** | **Per MJ_th_** |
| Agricultural land occupation | m^2^*a | 5.82E-03 | 8.99E-05 |
| Climate Change | kg CO_2_ eq. | 4.43E-02 | 3.30E-02 |
| Fossil fuel depletion | kg oil eq. | 1.18E-02 | 1.26E-02 |
| Freshwater ecotoxicity | kg 1.4-DB eq. | 6.31E-04 | 6.22E-05 |
| Freshwater eutrophication | kg P eq. | 3.89E-05 | 1.84E-06 |
| Human toxicity | kg 1.4-DB eq. | 2.30E-02 | 1.72E-03 |
| Ionising radiation | kg U235 eq. | 2.09E-02 | 8.06E-04 |
| Marine ecotoxicity | kg 1.4-DB eq. | 5.85E-04 | 5.97E-05 |
| Marine eutrophication | kg N eq. | 1.02E-05 | 1.52E-06 |
| Mineral resource depletion | kg Fe eq. | 1.27E-04 | 2.71E-04 |
| Natural land transformation | m^2^ | 4.26E-06 | 8.26E-06 |
| Ozone depletion | g CFC-11 eq. | -3.46E-06 | 5.19E-06 |
| Particulate matter formation | kg PM_10_ eq. | 5.35E-05 | 1.72E-05 |
| Photochemical oxidant formation | kg NMVOC | 7.89E-05 | 4.19E-05 |
| Terrestrial acidification | kg SO_2_ eq. | 1.30E-04 | 4.35E-05 |
| Terrestrial ecotoxicity | kg 1.4-DB eq. | -1.25E-06 | 4.05E-07 |
| Urban land occupation | m^2^*a | 2.34E-04 | 3.92E-05 |
| Water depletion | m^3^ | 3.78E-01 | 1.99E-02 |
