# Supplementary material for: Novel Miscanthus Germplasm-Based Value Chains: A Life Cycle Assessment
Source: Front Plant Sci. 2017 Jun 8;8:990. doi: 10.3389/fpls.2017.00990 (PMC5462955; doi:10.3389/fpls.2017.00990)
Supplement: Supplementary file 10 [file Table10.DOCX]

Table S10: Credits given for the by-product heat and for fermentation residues, which can be used as substitute for mineral fertilizers (utilization pathway 5)

| **Results per ha (Stuttgart)** | **Reference unit** | **Credits given** | |
| --- | --- | --- | --- |
|  |  | **Heat** | **Residues** |
| Agricultural land occupation | m^2^*a | 1.58 | 40.68 |
| Climate Change | kg CO_2_ eq. | 1371.37 | 1546.26 |
| Fossil fuel depletion | kg oil eq. | 545.30 | 223.20 |
| Freshwater ecotoxicity | kg 1.4-DB eq. | 1.87 | 10.66 |
| Freshwater eutrophication | kg P eq. | 0.06 | 0.33 |
| Human toxicity | kg 1.4-DB eq. | 45.06 | 482.38 |
| Ionising radiation | kg U235 eq. | 33.17 | 47.54 |
| Marine ecotoxicity | kg 1.4-DB eq. | 1.79 | 10.99 |
| Marine eutrophication | kg N eq. | 0.04 | 0.35 |
| Mineral resource depletion | kg Fe eq. | -4.34 | 101.76 |
| Natural land transformation | m^2^ | 0.36 | 0.19 |
| Ozone depletion | g CFC-11 eq. | 0.23 | 0.08 |
| Particulate matter formation | kg PM_10_ eq. | 0.31 | 2.34 |
| Photochemical oxidant formation | kg NMVOC | 0.85 | 3.67 |
| Terrestrial acidification | kg SO_2_ eq. | 0.27 | 6.97 |
| Terrestrial ecotoxicity | kg 1.4-DB eq. | 0.02 | 0.13 |
| Urban land occupation | m^2^*a | 1.41 | 22.76 |
| Water depletion | m^3^ | 777.54 | 1770.53 |
